# Efficiency of Xist-mediated silencing on autosomes is linked to chromosomal domain organisation

**DOI:** 10.1186/1756-8935-3-10

**Published:** 2010-05-07

**Authors:** Y Amy Tang, Derek Huntley, Giovanni Montana, Andrea Cerase, Tatyana B Nesterova, Neil Brockdorff

**Affiliations:** 1 MRC Clinical Sciences Centre, Faculty of Medicine ICSTM, Hammersmith Hospital, London, UK; 2 Morgan Building, Wellcome Trust Sanger Institute, Wellcome Trust Genome Campus, Hinxton, Cambridge, UK; 3 Centre for Bioinformatics, Division of Molecular Biosciences, Imperial College London, London, UK; 4 Department of Mathematics, Imperial College, London, UK; 5 Department of Biochemistry, University of Oxford, Oxford, UK

## Abstract

**Background:**

X chromosome inactivation, the mechanism used by mammals to equalise dosage of X-linked genes in XX females relative to XY males, is triggered by chromosome-wide localisation of a *cis*-acting non-coding RNA, Xist. The mechanism of Xist RNA spreading and Xist-dependent silencing is poorly understood. A large body of evidence indicates that silencing is more efficient on the X chromosome than on autosomes, leading to the idea that the X chromosome has acquired sequences that facilitate propagation of silencing. LINE-1 (L1) repeats are relatively enriched on the X chromosome and have been proposed as candidates for these sequences. To determine the requirements for efficient silencing we have analysed the relationship of chromosome features, including L1 repeats, and the extent of silencing in cell lines carrying inducible Xist transgenes located on one of three different autosomes.

**Results:**

Our results show that the organisation of the chromosome into large gene-rich and L1-rich domains is a key determinant of silencing efficiency. Specifically genes located in large gene-rich domains with low L1 density are relatively resistant to Xist-mediated silencing whereas genes located in gene-poor domains with high L1 density are silenced more efficiently. These effects are observed shortly after induction of Xist RNA expression, suggesting that chromosomal domain organisation influences establishment rather than long-term maintenance of silencing. The X chromosome and some autosomes have only small gene-rich L1-depleted domains and we suggest that this could confer the capacity for relatively efficient chromosome-wide silencing.

**Conclusions:**

This study provides insight into the requirements for efficient Xist mediated silencing and specifically identifies organisation of the chromosome into gene-rich L1-depleted and gene-poor L1-dense domains as a major influence on the ability of Xist-mediated silencing to be propagated in a continuous manner *in cis*.

## Background

Classical studies on X; autosome rearrangements have demonstrated that X inactivation propagates *in cis *from a single locus on the X chromosome, the X inactivation centre (Xic), and additionally highlighted that autosomal genes *in cis *with the Xic are inactivated less efficiently than normal X-linked genes [1-4]. This latter observation was suggested to be due to inefficient propagation or maintenance of X inactivation. More recently it has been shown that chromosome coating by X inactive specific transcript (Xist) RNA is the primary *cis*-acting trigger for X inactivation [5-7], and moreover that expression of Xist transgenes randomly integrated on mouse autosomes leads to coating and chromosome-wide silencing [8,9]. However, in other studies it has been shown that Xist RNA coating and associated silencing is compromised on autosomal chromatin in specific X; autosome rearrangements [10-14].

The relative insensitivity of autosomal genes to the X inactivation process led Gartler and Riggs to propose the idea of 'booster elements', sequences present on the X chromosome that evolved to facilitate the process of X inactivation [15]. Subsequently, Lyon noted that LINE-1 retrotransposons (L1s) are more highly represented on the X chromosome, and proposed that L1s may function as 'booster elements' [16]. L1s are an abundant dispersed repeat class that comprises close to 20% of the genome in mammals [17]. L1 enrichment on the X chromosome was substantiated by sequence analysis of mammalian genomes [18,19], and it was further shown that genes on the human X chromosomes that escape from X inactivation correlate negatively with the density of L1 elements [18-20]. Interestingly, methylation of LI elements is differentially regulated on the active and inactive X chromosome, the latter being dependent on the *de novo *DNA methyltransferase DNMT3B [21]. Consistent with these ideas a recent study found that intragenic L1 elements correlate with reduced expression of endogenous human genes [22].

In this study we have exploited inducible Xist transgenes located on different mouse chromosomes to carry out chromosome-wide analysis of Xist-mediated silencing on autosomes. Using bioinformatic and statistical analyses we then determined if any specific chromosome features predict the probability of a gene being silenced. The data demonstrate that genes located centrally in large gene-rich L1-depleted domains are relatively resistant to silencing and that conversely genes located in gene-poor domains enriched for L1 elements are inactivated more efficiently. We discuss these results in relation to models for Xist-mediated chromosome silencing.

## Results

### Xist-mediated silencing of genes on mouse chromosomes 3, 12 and 17

To identify *cis*-acting features associated with inefficient silencing on autosomes we carried out genome-wide analysis of gene expression in mouse embryonic stem (ES) cell lines expressing an autosomal doxycycline-inducible Xist transgene. Because autosomes in many instances have distinct domains with either high or low L1 density, in contrast to the X chromosome (see below), this strategy provided us with a basis to assess the contribution of L1s in Xist-mediated gene silencing.

**Figure 1:**
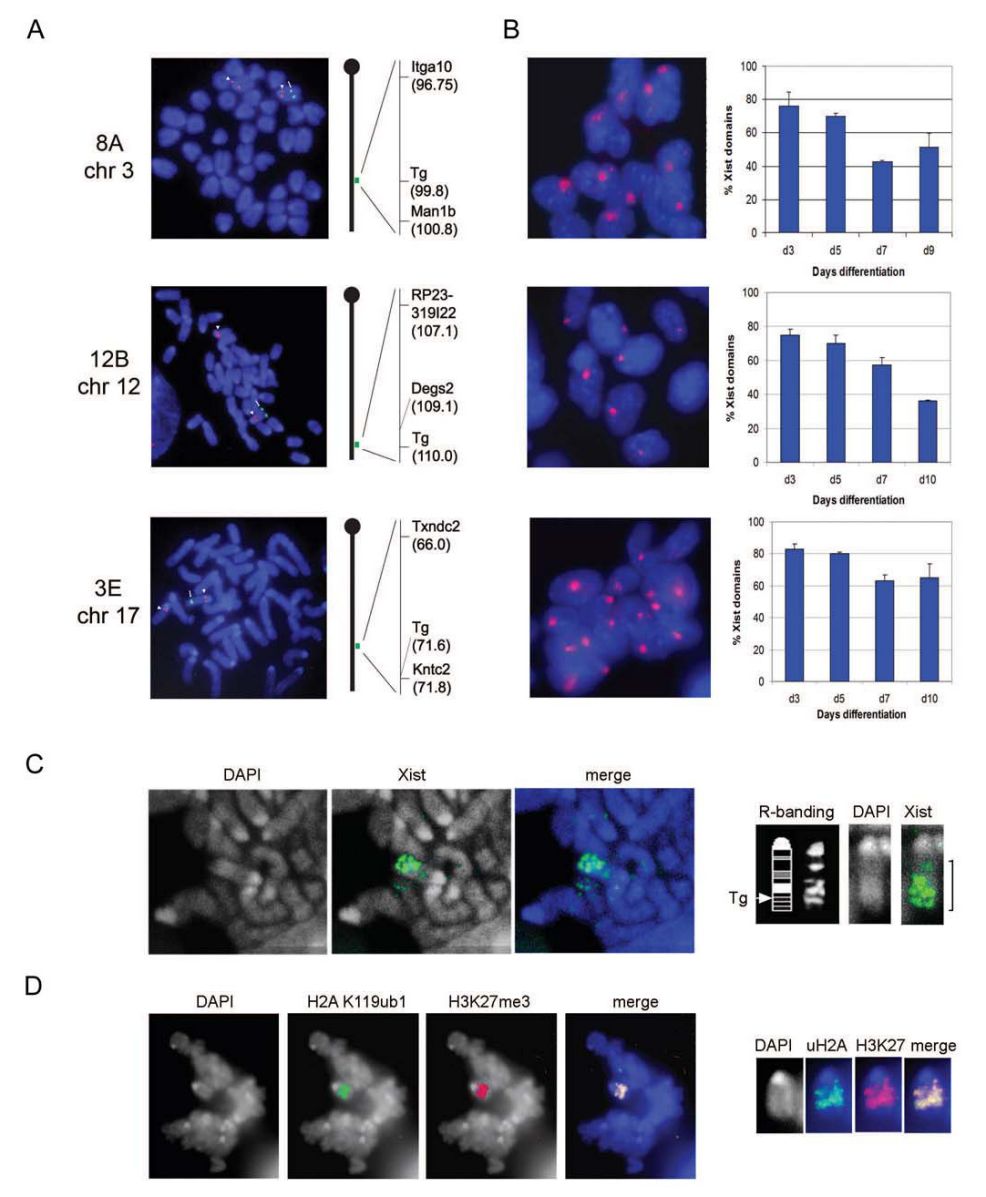
**Embryonic stem (ES) cell lines carrying autosomal-inducible Xist transgenes**. Three independent ES cell lines carrying inducible Xist transgenes on chromosomes 3 (8A), 12 (12B), and 17 (3E), were characterised in detail. **(a) **Panels illustrate example of DNA fluorescence in situ hybridisation (FISH) mapping of Xist transgene (green, arrow), and chromosome specific bacterial artificial chromosome (BAC) probe (red, arrowhead). DNA was counterstained with 4',6-diamidino-2-phenylindole (DAPI; blue). Maps illustrate fine mapping of each transgene (Tg) by DNA FISH, indicating position of nearest BAC probes in Mb. **(b) **RNA FISH analysis illustrating Xist RNA domains (red) in cells differentiated for 3 days in the presence of doxycycline. DNA was counterstained with DAPI (blue). Graphs illustrate percentage of cells with Xist RNA domains at different number of days (d) of differentiation. Error bars indicate variation from 2 independent determinations, scoring at least 100 cells in each case. **(c) **Example of RNA FISH of iXist in a mitotic cell from differentiating 3E cells (chromosome 17) illustrating Xist RNA bands along the length of the chromosome (right panels). **(d) **Immunofluorescence analysis of mitotic 3E cells illustrates chromosome-wide enrichment of Xist RNA dependent histone modifications H2AK119ub1 and H3K27 me3.

XY ES cell lines carrying Xist transgenes were derived essentially as described previously [9]. We focused our analysis on three independent cell lines, 8A, 12B and 3E, in which the transgene integration site was mapped to chromosomes 3, 12 and 17, respectively (Figure 1a). Xist induction in differentiating ES cells was assessed by RNA fluorescence in situ hybridisation (FISH) of interphase nuclei. In all three cell lines induced Xist RNA (iXist) formed discrete and robust nuclear domains in a large proportion (75% to 80%) of cells (Figure 1b). Analysis of differentiation time points revealed that the frequency of iXist domains diminishes as differentiation proceeds (Figure 1b), likely reflecting selection against cells that are functionally haploid for the transgene-bearing chromosome. iXist RNA, and also Xist dependent histone modifications were observed along the length of chromosomes carrying iXist transgenes. Examples for cell line 3E (chromosome 17 integration) are illustrated in Figure 1c, d.

Because only one of two alleles will be silenced in response to iXist expression the maximal downregulation for individual genes on Xist-bearing autosomes is 50%. Given the high efficiency of Xist induction in early stage differentiating cultures we considered that it should be possible to detect this level of change using gene expression microarrays. RNA samples were prepared from three or more biological replicates of differentiating ES cell cultures at 72 h either with or without doxycycline treatment. cRNA was prepared from RNA and hybridised to Affymetrix mouse whole genome 430 2.0 3' expression microarrays. Following preprocessing of data and statistical tests on differential expression, significantly upregulated or downregulated probe sets (false discovery rate <5%) were identified and then mapped to Ensembl genes http://www.ensembl.org/index.html. Genes that were not associated with any differentially expressed probe sets were categorised as showing no change.

**Figure 2:**
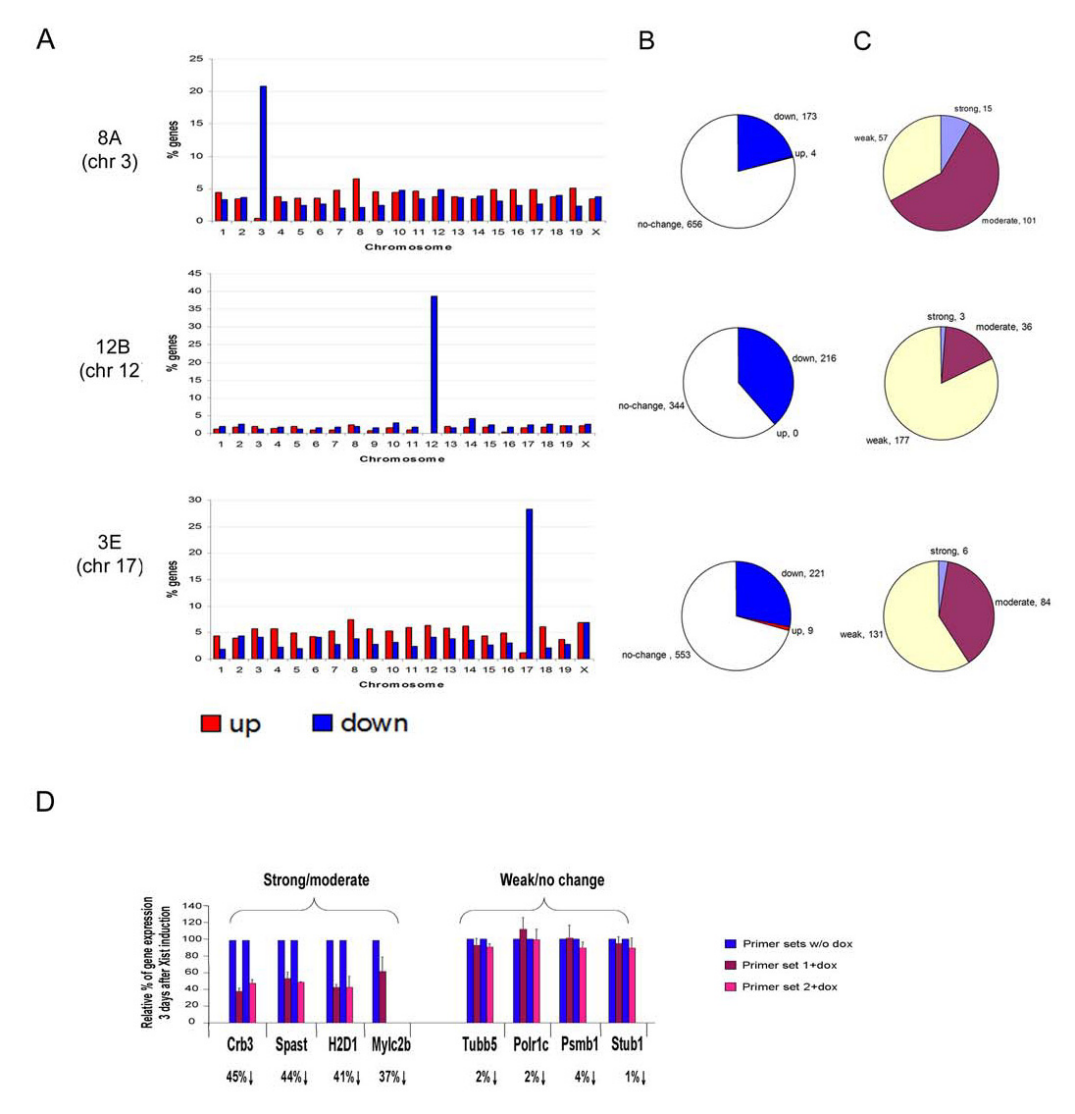
**Systematic analysis of gene expression in transgenic embryonic stem (ES) cell lines**. **(a) **The proportion of downregulated (blue) and upregulated (red) genes on all chromosomes following doxycyline treatment of transgenic cell lines as indicated. **(b) **Pie charts illustrating the proportion of downregulated (blue), upregulated (red) and no change (open) genes on the chromosome carrying the transgene, as indicated. **(c) **Pie chart illustrating the proportion of downregulated genes showing strong (>50%, blue), moderate (30% to 50%, red) and weak (<30%, yellow) downregulation, as indicated. **(d) **Quantitative PCR analysis of eight chromosome 17 genes showing strong/moderate or weak/no change in Affymetrix microarray data. Two different primer pairs were used for each gene (except Mylc2b). The average percentage reduction determined for probe sets by microarray analysis is shown below the gene symbols.

The distribution of differentially expressed probe sets assigned to Ensembl genes was analysed across all chromosomes. Significant changes in expression were predominantly associated with downregulated probe sets and a large proportion of these mapped to the chromosome carrying the iXist transgene (Figure 2a and http://web.bioinformatics.ic.ac.uk/geb/atang/). The proportion of genes downregulated on chromosomes 3, 12 and 17 was 21%, 39% and 28%, respectively (Figure 2b).

For each downregulated gene, silencing was further classified as strong (>50%), moderate (30% to 50%) or weak (<30%). Distribution of these classes varied from chromosome to chromosome but in all cases strongly downregulated genes were relatively infrequent (Figure 2c). A high proportion of weakly downregulated genes was observed on chromosome 12, and this is probably attributable to increased sensitivity of the analysis, which was based on five, rather than three replicate samples in the control and treated groups. It should be noted that the no change genes, which in all cases represented the largest class, include genes that are resistant to iXist-mediated silencing, and also genes for which the sensitivity of the assay is insufficient to detect downregulation. Assessment of gene expression levels by Affymetrix microarray analysis (Affymetrix, Santa Clara, CA, USA) was validated using quantitative (q)PCR analysis of selected genes showing moderate/strong or weak/no change (Figure 2d).

### Distribution of autosomal genes undergoing Xist-mediated silencing

To visualise the distribution of downregulated genes we used a software tool, the Genome Environment Browser (GEB) that was developed in house to facilitate analysis of high-throughput genomics data relative to chromosome features, notably L1 repeats [23]. GEB facilitates visualisation of the modular organisation of chromosomes into domains with high L1 density (HL1), enriched for relatively young full length L1s (FL-L1), and domains with low L1 density (LL1) that contain only fragmented or degenerated elements. Examples are illustrated in Additional files 1 and 2 and can be viewed dynamically by downloading GEB http://web.bioinformatics.ic.ac.uk/geb/atang/. Cytogenetic studies indicate that these domains broadly define chromosome G and R banding [24]. HL1 domains are often gene poor but are enriched for specific classes of genes, for example olfactory and vomeronasal receptor genes (Additional file 2). LL1 domains on the other hand are highly enriched for genes and also for CpG islands. The extent of HL1 and LL1 domains ranges from <500 Kb through to several Mb. The X chromosome is exceptional in having relatively high L1 and FL-L1 density throughout with only a few gene-rich LL1 domains that are in turn atypically small (Additional file 3).

**Figure 3:**
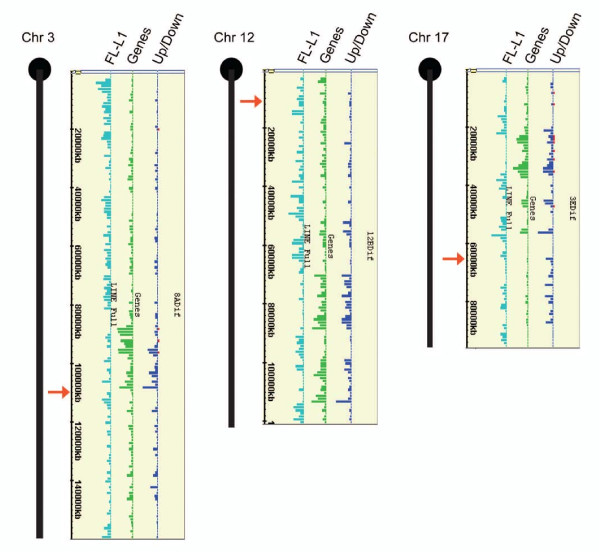
**Silencing propagates along the length of iXist transgene expressing autosomes**. The figure illustrates at 1 Mb resolution the distribution of downregulated (dark blue bars) and upregulated (red bars) genes on the transgene-bearing chromosomes for cell lines 8A, 12B and 3E, respectively. The height of the bar indicates the frequency of genes per Mb (maximum height in the different cell lines corresponds to 12-14 genes/Mb). Tracks showing the frequency of genes (green), and, full-length L1 elements (light blue), are also shown. The approximate position of the transgene integration site is indicated with a red arrow.

As illustrated in Figure 3, downregulated genes were located along the entire length of the transgene-bearing chromosomes, chromosome 3, 12 and 17 regardless of their overall length (approximately 160, 120 and 100 Mb, respectively). Their distribution broadly mirrors overall gene density and distance from the transgene integration site does not appear to affect the probability of silencing. These results substantiate RNA FISH data indicating that Xist RNA traverses the entire length of the transgene-bearing chromosome (Figure 1c, d).

**Figure 4:**
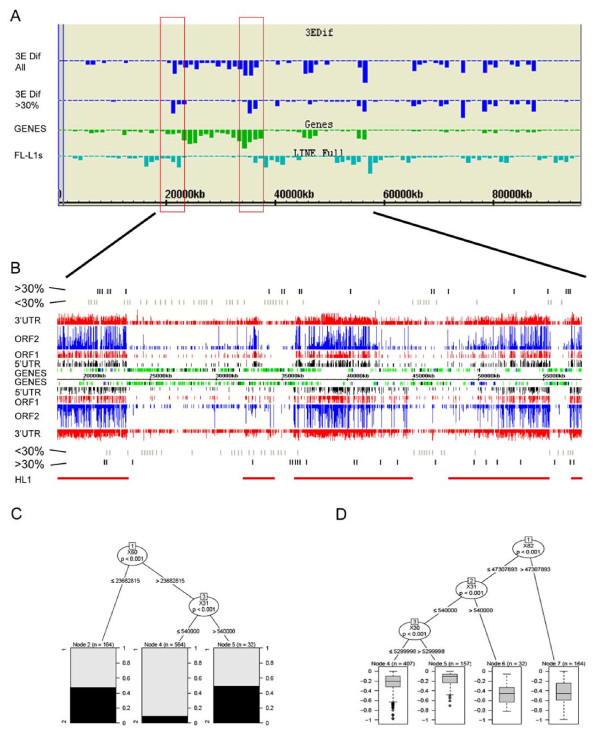
**Genes with strong downregulation cluster within and flanking domains with high L1 density on chromosome 17**. **(a) **Genome Environment Browser (GEB) histogram display showing distribution of chromosome 17 genes with ≥ 30% downregulation compared with all downregulated genes. Gene density (green) and full-length L1 (FL-L1) density (light blue) are also shown. Boxes highlight >30% genes enriched in high L1 domains flanking a large gene-rich region. **(b) **GEB detail view of Mb 18-58. High L1 density (HL1) domains are visualised as regions with high density of vertical lines (L1 5' untranslated region (UTR), open reading frame (ORF)1, ORF2 and 3' UTR) and are highlighted for illustrative purposes with horizontal red lines. Genes are represented as green boxes. Those showing >30% and <30%downregulation are blue and black, respectively. Downregulated classes are also highlighted by black (>30%) and grey (<30%) bars above and below the GEB display. **(c) **Classification tree comparing 20% most downregulated genes on chromosome 17 with all other genes. The first split divides the chromosome into two, proximal and distal of 56 Mb, based on the distance between the gene and a unique L1VL4 element at 80.4 Mb (×60). On the right side of the tree, representing the proximal 56 Mb, the model suggests genes located in HL1 domains larger than 0.54 Mb (×31) had a much higher probability of being silenced. **(d) **Regression tree for chromosome 17 highlights equivalent L1 domain related features. At the terminal nodes, the lower the expression values, the stronger the gene silencing effect. The tree defines that to remain active the gene should be (1) within the first 56 Mb of the chromosome (node 1 split, ×82), (2) associated with a relatively small HL1, or no HL1 at all (node 2 split, ×31), and (3) associated with a low L1 density (LL1) domain at least approximately 5.3 Mb large (node 3 split, ×30). Two large LL1 domains on chromosome 17 fit this description. Y-axis scale on box and whisker plots shows expression level of genes relative to untreated control in log2 scale.

Whilst the distribution of all downregulated genes was seen to mirror overall gene density, analysis of only moderate/strong (>30%) downregulation revealed a distinct and striking pattern. Specifically, using the chromosome 17 dataset we observed an apparent enrichment of these genes both within and immediately flanking L1 enriched domains (Figure 4a). This is further illustrated in Figure 4b showing a GEB detailed view of Mb 38-58 on chromosome 17. The frequency of genes showing <30% downregulation (grey bars) correlates with overall gene density whereas genes showing >30% (black bars) are on the whole located within or close to the borders of L1 enriched domains.

### Xist-mediated silencing on autosomes is linked to chromosomal domain organisation

To confirm and extend our observations we carried out a data-driven, multivariate statistical analysis to determine the genomic feature(s) that best explain the variation in gene expression in response to iXist-mediated silencing. No prior hypothesis was injected into the models, although with the proposed role of L1 in iXist-mediated silencing, several L1-related features were measured. A set of 83 candidate features was included and comprised measures of intrinsic characteristic of a gene (gene size, intron size), the distance from transgene integration site, the local density of repetitive elements (L1, short interspersed nuclear elements (SINEs) or long terminal repeats (LTRs)) at various distances up/downstream of a gene, the positioning of a gene with respect to defined HL1 or LL1 domains, and the distance from the gene to its nearest FL-L1 element of different subfamilies. Additional file 4 provides the full list of 83 features. Coordinates of domains defined as HL1 and LL1 for chromosomes 3, 12 and 17 are listed in Additional file 5.

Two different modelling approaches, classification and regression trees, were applied. Classification trees compare specified deciles, for example the 10% most strongly silenced versus all other genes, and define the features that best predict these classes. Regression trees do not rely on a prior clustering of genes into groups, and provide rules that best describe the observed distribution of fold changes. In the case of chromosome 17 both approaches clearly identify L1 domain features as the primary determinant of silencing efficiency. An example of a classification tree comparing the top 20% most strongly downregulated genes with all the remaining genes is illustrated in Figure 4c. The top feature selected by this and all the classification models results in the division of chromosome 17 into two regions, one of them encompassing one or both large LL1 domains discussed above, implying that the distribution of fold change differs in the two regions of the chromosome. After the first split in the trees, chromosomal domain-related features were chosen by the models as being statistically significant, formulating rules which suggest that genes located in large LL1 domains are less susceptible to iXist-mediated silencing (and vice versa for genes located in large HL1 domains). Similar rules were formulated by classification trees for chromosome 17 that consider downregulated genes of other levels of severity (Additional file 6). Similarly, the regression tree for chromosome 17 first selected a feature that divides the chromosome into two distinct regions, based on the location of the large LL1 domains, with the following splits down the tree predicting gene silencing in large HL1 domains or insensitivity to gene silencing in large LL1 domains (Figure 4d).

The importance of chromosomal domain structure in dictating silencing efficiency was further supported by modelling the chromosome 3 dataset. Here, both classification and regression approaches determined that genes located within LL1 domains >1.3 Mb in size are relatively resistant to iXist-mediated silencing (Additional file 7). For the chromosome 12 dataset classification trees using a subset of features and regression trees identified low SINE repeat density but not L1 domain related features with silencing efficiency (Additional files 8 and 9). Because SINEs are distributed reciprocally to L1s, being localised to gene-rich domains, this result is not entirely inconsistent with those obtained for chromosomes 3 and 17. Additionally it should be noted that L1 domain modularity is less clear cut on some autosomes. Neither chromosome 3 nor chromosome 12 have large (>5 Mb) L1-depleted gene-rich domains, and moreover gene-rich domains on chromosome 12 are regularly interrupted by short regions with high L1 density (examples can be viewed dynamically by downloading GEB from http://web.bioinformatics.ic.ac.uk/geb/atang/).

## Discussion

In this study we have exploited inducible Xist transgenes to analyse Xist-mediated gene silencing in an autosomal context. Inactivation of genes on transgene-bearing chromosomes was assayed by microarray analysis, and bioinformatic and statistical approaches were applied to determine which chromosomal features best predict Xist-mediated silencing. The results demonstrate that genes located in the core of large gene-rich domains with a low density of L1 repeats are relatively resistant to silencing and conversely that genes located within domains with a high density of L1 repeat elements are inactivated more efficiently. This could provide a basis for the observed selection in favour of L1 enrichment on the eutherian X chromosome [25], with the acquisition of L1 elements fragmenting large gene-rich domains present on the ancestral X chromosome, leading to increased efficiency of dosage compensation for the entire chromosome.

Of the chromosome features analysed in this study, chromosomal domain organisation as defined by large regions with predominantly high or low L1 density was selected as providing a strong association with efficiency of silencing. As suggested above, the contribution of these domains to the efficiency of silencing may only be evident on those chromosomes that show a highly modular organisation of large HL1 and LL1 domains. It is important to note that these findings do not implicate L1 elements directly in Xist mediated silencing as features of HL1 domains other than L1 elements may account for their association with increased silencing efficiency. Moreover the results may equally well be interpreted to indicate that it is features of LL1 domains, for example gene density or SINE density that confers resistance to Xist mediated silencing. It is also possible that beyond chromosome domain organisation other untested features of chromosome organisation contribute to reduced efficiency of silencing on autosomes relative to the X chromosome.

Xist RNA spreading occurs across the entire length of the chromosome in all three cell lines used in this study, similar to results obtained in previous studies on autosomal Xist transgenes [8,9]. These observations have been broadly interpreted to indicate that Xist efficiently silences autosomes. However, by carrying out chromosome-wide analysis of gene expression we demonstrate that there are marked discontinuities in silencing, notably resistance to silencing in large gene-rich LL1 domains. Thus spreading of Xist-mediated silencing should be viewed not as a linear and continuous process but rather as a series of hops and jumps.

Evidence for discontinuous silencing of autosomal chromatin has been documented previously. For example in Is1ct, an insertion of a region of chromosome 7 into the X chromosome, there is evidence that X inactivation 'skips' over the chromosome 7 material [2,3]. Similarly in a patient with an unbalanced X; A translocation, expression analysis of individual loci demonstrated discontinuous silencing of autosomal genes *in cis *with the inactive X [13]. In addition there are a number of genes on the X chromosome that escape X inactivation and these are often flanked on both sides by genes subject to X inactivation [26]. In the case of Is1ct discontinuous inactivation is at least in part attributable to 'spread and retreat' of X inactivation where chromosome 7 genes are initially silenced and then progressively reactivated [3]. In this study discontinuous inactivation is apparent when Xist is first expressed, demonstrating that escape from silencing can also occur as a result of an inherent resistance to Xist-mediated silencing at specific loci or domains.

Although chromosome-wide spreading of Xist RNA occurs in most cases that have been analysed, there is at least one example, the X; A translocation T(X;4)37H, where autosomal chromatin acts as a boundary that blocks spreading both of Xist RNA and associated chromatin modifications [10,11]. Interestingly this block in spread correlates with the presence of an exceptionally large (approximately 20 Mb) gene-rich LL1 domain located on the autosomal product at the translocation breakpoint [11]. With this in mind we speculate that LL1 domains generally resist Xist-mediated silencing but that this effect is more pronounced in larger LL1 domains and that beyond a specified size, LL1 domains inhibit both further spreading of Xist RNA and associated silencing.

Paradoxically studies analysing Xist RNA localisation on metaphase chromosomes demonstrate enrichment of the RNA over R bands (gene-rich domains) and exclusion from G bands (high L1 density) and constitutive heterochromatin [10,27], the opposite of what might be predicted from this study. It seems unlikely on this basis that L1 elements function directly as landing sites that enhance spread of Xist RNA. With this in mind we envisage two possible scenarios whereby L1 domain organisation may influence Xist-mediated silencing. First, HL1 domains may concentrate factors involved in DNA methylation or the RNAi response, both of which are implicated in silencing of L1 transcription [28,29]. This in turn may facilitate silencing by Xist. Second, recent studies suggest that Xist creates a silencing compartment in the interphase nucleus into which genes on the X chromosome are progressively recruited [30], possibly facilitated by the scaffold attachment factor SATB1 [31]. There is evidence that L1 domain organisation has a role in the spatial organisation of chromosomes [32,33], and this in turn could modulate the likelihood that nearby genes are recruited into the Xist silencing compartment.

## Conclusions

This study demonstrates that domain organisation of mouse autosomes as defined by L1 density influences the efficiency of gene silencing in response to expression of Xist transgenes. Our findings provide a possible explanation for selection favouring accumulation of L1 elements on the X chromosome in placental mammals.

## Methods

### iXist transgene and cell lines

Xist cDNA (17.9 kb) covering exons 1-8 was cloned in pBluescript (Stratagene, La Jolla, CA, USA) in three steps. Briefly, the 6.7 kb *Eco*RI-*Bam*HI genomic DNA fragment from 129 strain background covering exons 7-8 and intron 7 was cloned into pBluescript, giving pBSX1. In the second step, the middle region of the cDNA (2.5 kb) was amplified by reverse transcription (RT)-PCR in two contiguous fragments of 1.2 kb (with AT1/VMEX5 primers) and 1.6 kb (with SX7/AT2 primers) with a 0.3 kb overlapping region (as well as a common *Pvu*II site in exon 5), using PGK-strain kidney cDNA as PCR template. The RT-PCR products were cloned into pBSX1 by coligation, using the *Pvu*II and *Eco*RI sites at exons 5 and 7, respectively, giving pBSX5. Finally, an 8.9 kb *Sac*II-*Pac*I exon 1 genomic DNA fragment (also from 129 strain background) was cloned into pBSX5 at the *Pac*I site, giving pBSXist. Primer sequences: AT1:5'-aaatttgagaacatgtcaagtcgc-3'; AT2: 5'-caccaatagcaaagagacacaaaat-3'; SX7: 5'-acgatccctaggtggagat-3'; VMEX5: 5'-ggcatgagtagggtagcagt-3'. To create the Tet-responsive Xist transgene construct pTREXist, Xist cDNA was released from pBSXist by *Psp*OMI-*Cla*I digestion, and cloned into *Not*I-*Cla*I-digested pTRE-tight vector (Takara Bio Europe/Clontech, France).

To establish an inducible Xist transgenic ES cell line, pTREXist vector and a PGKneo cassette were coelectroporated into puromycin-resistant 129 strain XY ES cells carrying a reverse-tet transactivator (rtTA), where the pR26/P'nlsrtTA construct enclosing rtTA (gift from A Wutz, Wellcome Trust Centre for Stem Cell Research, Cambridge, UK) has been targeted initially into the constitutively active ROSA26 locus [34,35] as described previously [9]. To test the efficiency of transgene induction doxycycline (1 μg/ml) was added to the medium and 24 h later the cells were screened for transgene expression by RT-PCR across Xist exons 4 and 5, exploiting the *Hin*dIII polymorphism introduced when the transgene was cloned.

### Microarray analysis

Details of cell culture, sample preparation and hybridization for microarray analysis have been deposited in ArrayExpress http://www.ebi.ac.uk/microarray-as/ae/ under experiment accessions E-MEXP-2086 (chromosome 3), E-MEXP-2087 (chromosome 17) and E-MEXP-2088 (chromosome 12). Four strong/moderate downregulated genes and four weak/no change genes were selected for validation of microarray data. Downregulated genes were Crb3 (ENSMUSG00000044279), Spast (ENSMUSG00000024068), H2D1 (ENSMUSG00000073411) and Mylc2b (ENSMUSG00000034868); genes without change in expression selected for analysis were Tubb5 (ENSMUSG00000001525), Polr1c (ENSMUSG00000067148), Psmb1 (ENSMUSG00000014769) and Stub1 (ENSMUSG00000039615). Primer information and conditions for quantitative RT-PCR are available on request from the authors.

### Statistical analysis

The preprocessing and statistical analysis of the microarray data was performed as follows. For each cell line, probe hybridisation intensity data from all arrays were preprocessed and log2 transformed using the Robust Multichip Average (RMA) algorithm [36] in the 'affy' R/Bioconductor package http://www.bioconductor.org/. For cell lines 8A (chromosome 3) and 3E (chromosome 17) where expression data were available from ES cell samples, a 'no expression' baseline was estimated per cell line by taking the median expression value of approximately 100 probe sets mapped to genes known from previous experimental work to be normally silent in ES cells, and probe sets with expression values below this baseline in all samples were discarded. Data from cell line 12B (chromosome 12) were not filtered because expression data from ES cells were not available. For each probe set, the fold change in expression was calculated by subtracting the mean of control samples from the mean of treated samples. Detection of differential expression was carried out by using linear models and specifically the empirical Bayes methods [37] implemented in the R/Bioconductor package 'limma'. Multiple-testing correction was carried out in order to control the false discovery rate (FDR) using the methods of Benjamini and Hochberg [38] as implemented in the R/Bioconductor package 'multtest'. Combining differential expression data and probe set mapping data from Ensembl (version 46.36 g), up/downregulated probe sets were defined as those which were mapped to unique locations in the genome and with a FDR ≤ 5%. Downregulated genes were defined as those represented by at least one downregulated probe set and no upregulated probe set at FDR ≤ 5%. The expression value of a downregulated gene was calculated as the average of all associated downregulated probe sets. See Additional file 10 for further details.

Statistical modelling was used to determine whether there was a specific subset of genomic features that explained the observed patterns of gene expression. We employed non-parametric methods in order to model the conditional distribution of gene expression given the genomic features while also determining the importance of each feature and how the salient features interact with each other. Classification and regression trees (CART) models were fit to the experimental data because of their ability to model complex relationships between features, possibly measured on different scales, and deliver compact statistical representations or rules that can be easily visualized and interpreted. Parameter estimation was carried out using the recently developed conditional inference framework for fitting tree-based models based on permutation tests. This framework allowed us to perform feature selection using hypothesis testing procedures. See Additional file 10 for further details.

## Competing interests

The authors declare that they have no competing interests.

## Authors' contributions

YAT carried out most of the experimental work, contributed to the bioinformatic and statistical analysis and to the study design and manuscript preparation. DH and GM contributed to the bioinformatic and statistical analysis and manuscript preparation. AC contributed to the experimental work and manuscript preparation. TBN and NB contributed to the study design and manuscript preparation. All authors read and approved the final manuscript.

## Supplementary Material

Additional file 1 **Examples of L1 domain organisation**. **(a) **Genome Environment Browser (GEB) histogram plot of mouse chromosome 4 illustrating the frequency of genes (green bars), CpG islands (lilac bars), full length L1 (FL-L1) elements (light blue bars), L1 homology sequences (yellow bars) and short interspersed nuclear elements (SINEs, grey bars) within 1-Mb intervals. The 25 Mb regions highlighted by a red box are shown in GEB detailed view in (b). **(b) **The 25 Mb region 1 (top panel) with boxed areas illustrating small (left) and moderate (right) sized gene-rich domains that are depleted for FL-L1. Note that gene density (green boxes) is higher in these domains. The 25 Mb region 2 (bottom panel) illustrates a large low L1 density (LL1) domain (approximately 22 Mb) flanked on each side by a small high L1 density (HL1) domain. Click here for file

Additional file 2 **Olfactory receptor (OR) gene clusters localise to high L1 density (HL1) domains**. **(a) **Top panel: Genome Environment Browser (GEB) histogram display for mouse chromosome 9 illustrating one of the largest OR clusters in the mouse genome (marked by a red box), showing high gene density, low CpG island density and high full length L1 (FL-L1) density. Red arrows indicate other gene-rich regions on the chromosome that, as is generally the case, do not show FL-L1 enrichment. The lower panel shows the GEB detailed view of the OR cluster, black dotted lines marking the boundaries. **(b) **GEB detailed view illustrating a 6-Mb region covering the major histocompatibility complex (MHC) and an OR cluster on mouse chromosome 17 (34.0-40.0 Mb). Boxed areas mark the boundaries of the gene clusters. The OR genes and the MHC are in contrasting HL1 and low L1 density (LL1) domains, respectively. **(c) **GEB detailed view illustrating a 6-Mb region covering *Hist1 *histone (H) and vomeronasal receptor (VR) domains on mouse chromosome 13 (19.5-25.5 Mb). Boxed areas mark the boundaries of the gene clusters. Histone gene clusters are L1 depleted whereas VR, like OR, are in an HL1 domain. **(d) **Scatter plots illustrating L1 density of OR gene clusters in mouse and human (blue). *Hist1 *histone clusters are included for comparison (red). Click here for file

Additional file 3 **Unique organisation of high L1 density (HL1) and low L1 density (LL1) domains on the mouse X chromosome**. **(a) **Genome Environment Browser (GEB) histogram display of features on the mouse X chromosome as described for Additional file 1. Boxed 25 Mb regions 1-4 that have relatively high gene density are shown as GEB detailed view in (b). **(b) **Detailed views of 25 Mb domains. Note that LL1 regions in which genes are concentrated are very small compared with typical autosomes and overall L1 density is high across the entire chromosome. Click here for file

Additional file 4 **Supplementary table 1**. File contains a list of features used in multivariate analysis. Click here for file

Additional file 5 **Supplementary table 2**. File contains Ensembl coordinates (version 46.36 g) for the position of high L1 density (HL1) and low L1 density (LL1) domains on chromosomes 3, 12 and 17. Click here for file

Additional file 6 **Classification trees for downregulated genes on chromosome 17**. Classification trees comparing **(a) **10% most downregulated genes on chromosome 17 with all other genes. The first split divides the chromosome into two parts, proximal and distal of 35 Mb, based on the distance between the gene and a unique Lx5 element at 9.5 Mb (×82). The left side then shows that for the proximal 35 Mb of the chromosome downregulation is associated with location within a high L1 density (HL1) domain truncated by 500 kb at both ends (×37 >0). For genes distal to 35 Mb downregulation is either associated with location in a low L1 density (LL1) domain less than 1.3 Mb in size (×30), or in larger LL1 domains associated with L1 density downstream of a gene (×25) >3.7%. For trees comparing **(b) **30%, **(c) **40% and **(d) **50% most downregulated genes on chromosome 17 with all other genes, the first split from the top node divides the chromosome into two parts, proximal and distal of approximately 70 Mb, based on the distance between the gene and the nearest full-length (FL) L1_Mus2 element (×44) which can be found at four locations on chromosome 17 (20.7, 31.6, 41.2 and 54.5 Mb). In the proximal 70 Mb of the chromosome, the models suggest that genes less susceptible to silencing are located centrally in large LL1 domains, not associated with large HL1s, and/or surrounded by a low (≤ 12.77%) local L1 density. ×5, overlaps (1) or not (0) with an HL1 domain; ×25, L1 density 100 kb upstream of the gene; ×31, size of HL1 domain with which the gene is associated; ×33 and ×34 whether a gene overlaps (1) or not (0) with a core LL1 domain (LL1 truncated by 250 kb or 500 kb, respectively, at its proximal and distal boundaries). Y-axis denotes probability of a gene being silenced (black fill). Click here for file

Additional file 7 **Genes located in relatively large low L1 density (LL1) domains on chromosome 3 have a lower probability of being silenced**. This effect was seen across the first five deciles, in classification trees comparing **(a) **10%, **(b) **20%, **(c) **30%, **(d) **40% and **(e) **50% most downregulated genes against all other genes. For every decile, the tree estimated from the data only contained one split, which is either based on a LL1 domain size measure (×30), or on the location of the gene with respect to large LL1 domains (×33 and ×34). Generally, genes located in LL1 domains >1.31 Mb in size or those associated with 'core' LL1 regions (×33 or ×34 >0) have approximately 50% less chance of being silenced. Additional files 4 and 9 give further details on feature definition. Click here for file

Additional file 8 **Lower local short interspersed nuclear elements (SINE) density may be associated with gene silencing on chromosome 12**. Classification trees for chromosome 12 data identified a reciprocal relationship between iXist mediated silencing and SINE density. The effect was only observed when we compared the 10% **(a) **and 20% **(b) **most strongly downregulated genes on the chromosome to all other genes. ×26, SINE density in the 100 kb region upstream of the gene. Click here for file

Additional file 9 **Regression trees for chromosome 12**. **(a) **The first split (×80) measures the distance between the gene to the nearest full length (FL) Lx4B element (which is uniquely located at approximately 6.5 Mb), followed by the second split (×53) which records the orientation of the nearest FL L1 Md_F2 to the gene (1 = upstream of the gene, -1 = downstream). (There are 35 copies of FL L1 Md_F2 on chromosome 12 altogether.) The regression tree suggests that the gene silencing effect would be the strongest for a given gene not more than 18.89 Mb away from the FL Lx4B element, (in other words, in the proximal 25.5 Mb of the chromosome), with the nearest FL L1 Md_F2 element being downstream of the gene. **(b) **When estimated on a subset of genomic features, the regression tree modelled the gene silencing effect differently. Here the gene silencing effect is the strongest for genes with a relatively low local SINE density (≤ 14.5%) and not associated with the core of a large low L1 density (LL1) domain (×34 = 0). Note that genes located in the centre of a large LL1 domain have a median downregulation level of just 10% (node 4 of the tree), which is a very mild effect. Additional file 4 gives details on genomic feature definition. Click here for file

Additional file 10 **Supplementary methods**. File includes supplementary methods for RNA and DNA fluorescence in situ hybridisation (FISH) analysis, immunofluorescence, detailed description of statistical analysis, genomic data analysis and supplementary references. Click here for file
