# Supplementary figures and images for: Efficiency of Xist-mediated silencing on autosomes is linked to chromosomal domain organisation
Source: Epigenetics Chromatin. 2010 May 7;3:10. doi: 10.1186/1756-8935-3-10 (PMC2873326; doi:10.1186/1756-8935-3-10)

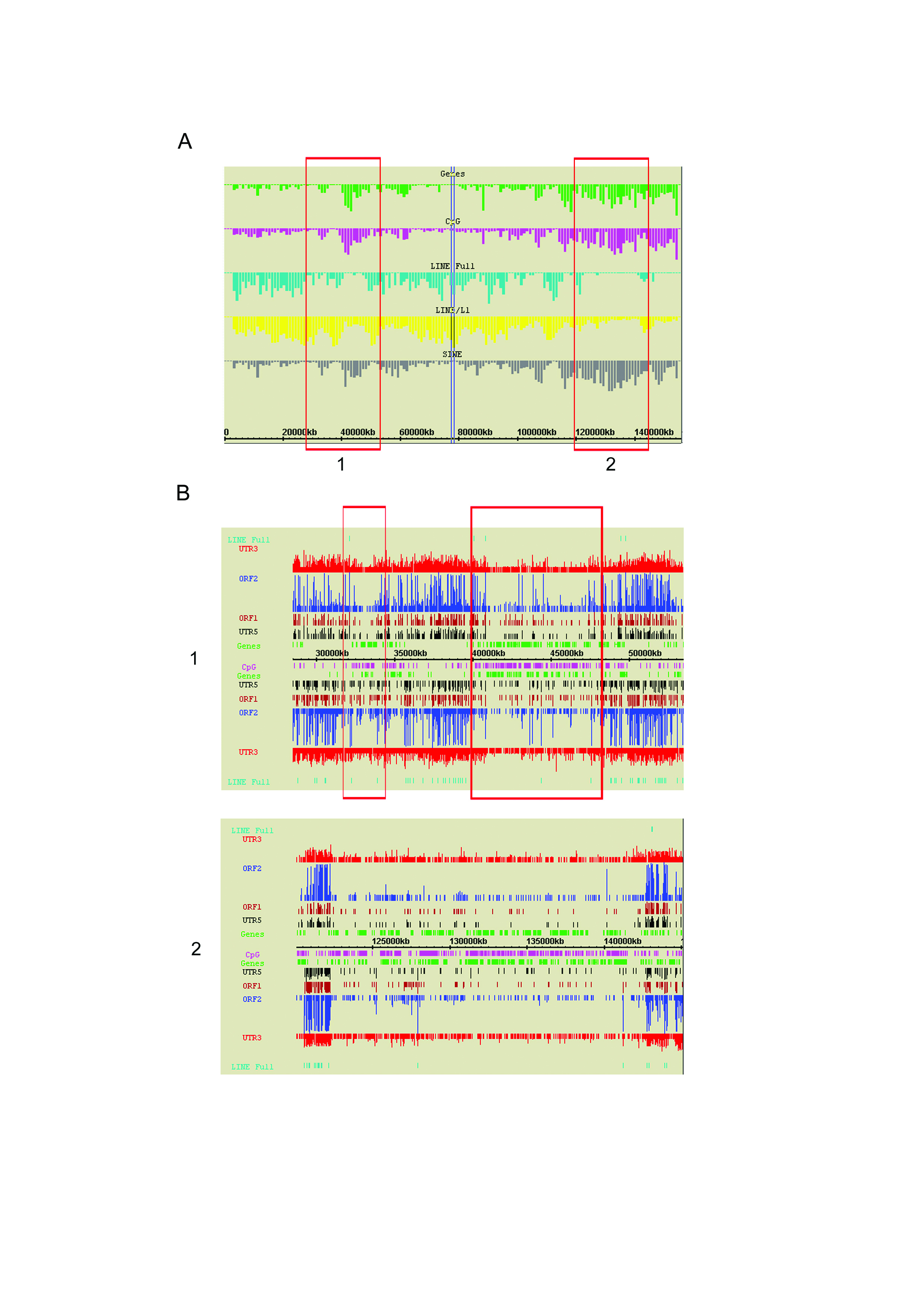

Supplement: Additional file 1 — Examples of L1 domain organisation. (a) Genome Environment Browser (GEB) histogram plot of mouse chromosome 4 illustrating the frequency of genes (green bars), CpG islands (lilac bars), full length L1 (FL-L1) elements (light blue bars), L1 homology sequences (yellow bars) and short interspersed nuclear elements (SINEs, grey bars) within 1-Mb intervals. The 25 Mb regions highlighted by a red box are shown in GEB detailed view in (b). (b) The 25 Mb region 1 (top panel) with boxed areas illustrating small (left) and moderate (right) sized gene-rich domains that are depleted for FL-L1. Note that gene density (green boxes) is higher in these domains. The 25 Mb region 2 (bottom panel) illustrates a large low L1 density (LL1) domain (approximately 22 Mb) flanked on each side by a small high L1 density (HL1) domain. [file 1756-8935-3-10-S1.TIFF]

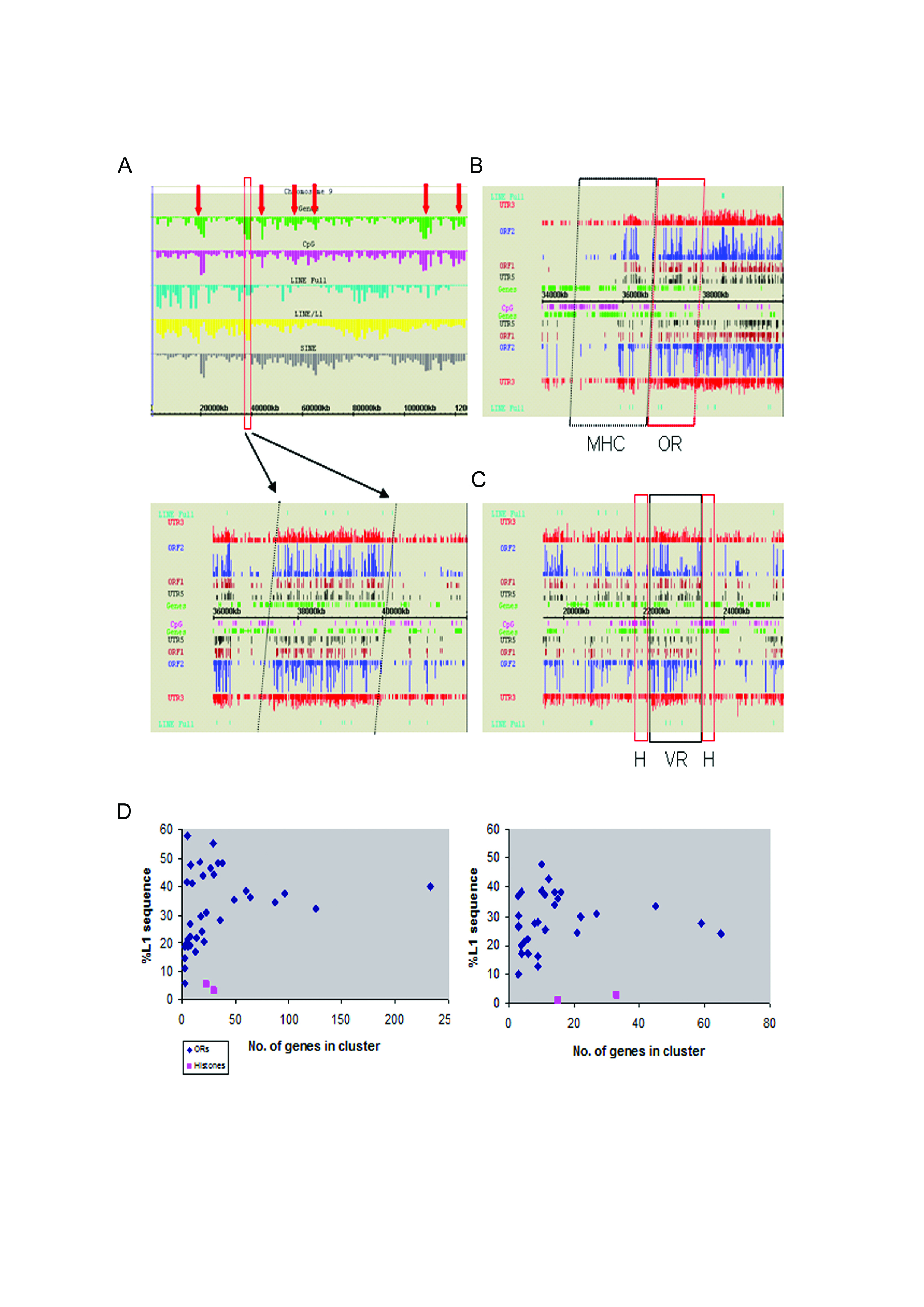

Supplement: Additional file 2 — Olfactory receptor (OR) gene clusters localise to high L1 density (HL1) domains. (a) Top panel: Genome Environment Browser (GEB) histogram display for mouse chromosome 9 illustrating one of the largest OR clusters in the mouse genome (marked by a red box), showing high gene density, low CpG island density and high full length L1 (FL-L1) density. Red arrows indicate other gene-rich regions on the chromosome that, as is generally the case, do not show FL-L1 enrichment. The lower panel shows the GEB detailed view of the OR cluster, black dotted lines marking the boundaries. (b) GEB detailed view illustrating a 6-Mb region covering the major histocompatibility complex (MHC) and an OR cluster on mouse chromosome 17 (34.0-40.0 Mb). Boxed areas mark the boundaries of the gene clusters. The OR genes and the MHC are in contrasting HL1 and low L1 density (LL1) domains, respectively. (c) GEB detailed view illustrating a 6-Mb region covering Hist1 histone (H) and vomeronasal receptor (VR) domains on mouse chromosome 13 (19.5-25.5 Mb). Boxed areas mark the boundaries of the gene clusters. Histone gene clusters are L1 depleted whereas VR, like OR, are in an HL1 domain. (d) Scatter plots illustrating L1 density of OR gene clusters in mouse and human (blue). Hist1 histone clusters are included for comparison (red). [file 1756-8935-3-10-S2.TIFF]

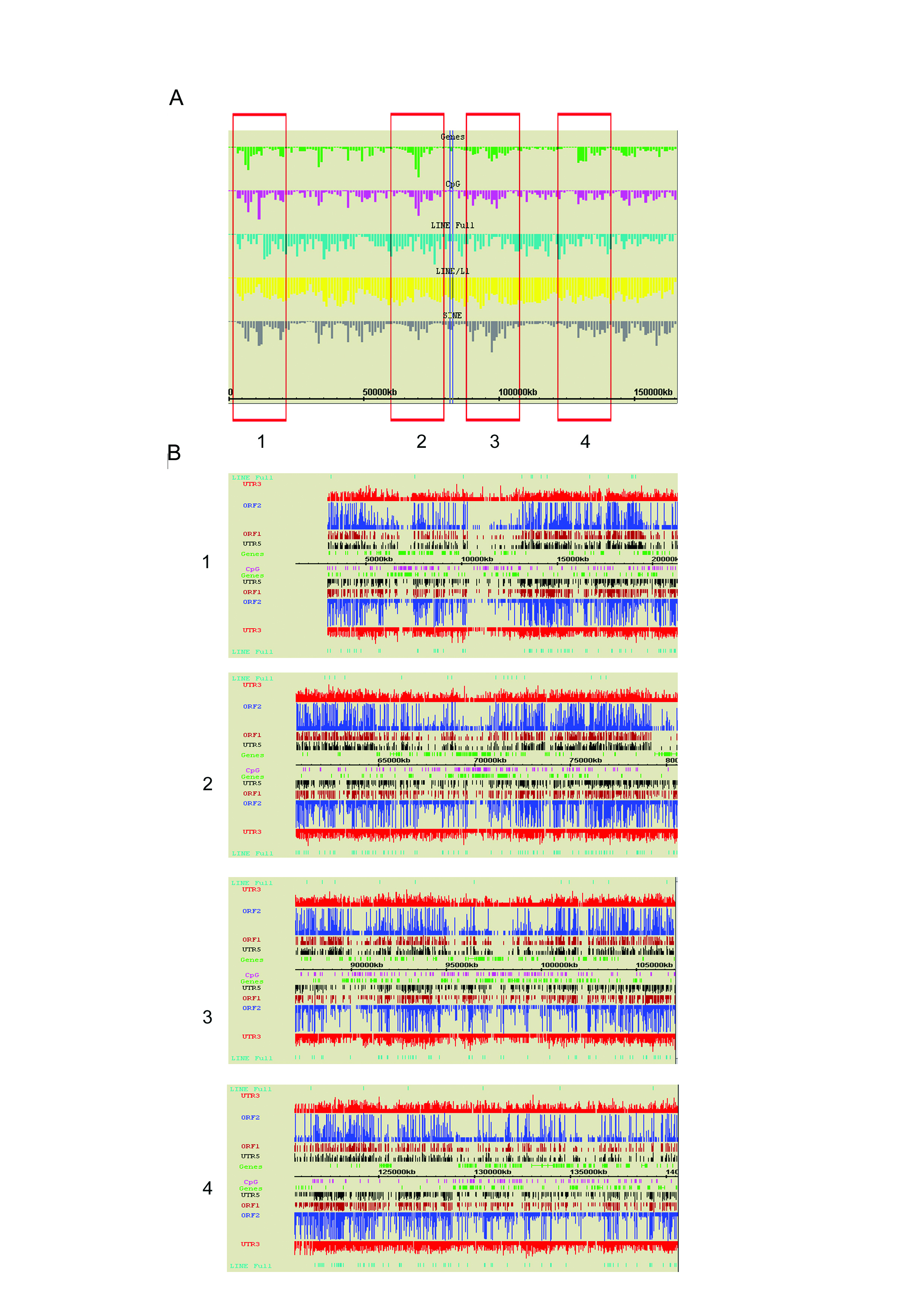

Supplement: Additional file 3 — Unique organisation of high L1 density (HL1) and low L1 density (LL1) domains on the mouse X chromosome. (a) Genome Environment Browser (GEB) histogram display of features on the mouse X chromosome as described for Additional file 1. Boxed 25 Mb regions 1-4 that have relatively high gene density are shown as GEB detailed view in (b). (b) Detailed views of 25 Mb domains. Note that LL1 regions in which genes are concentrated are very small compared with typical autosomes and overall L1 density is high across the entire chromosome. [file 1756-8935-3-10-S3.TIFF]

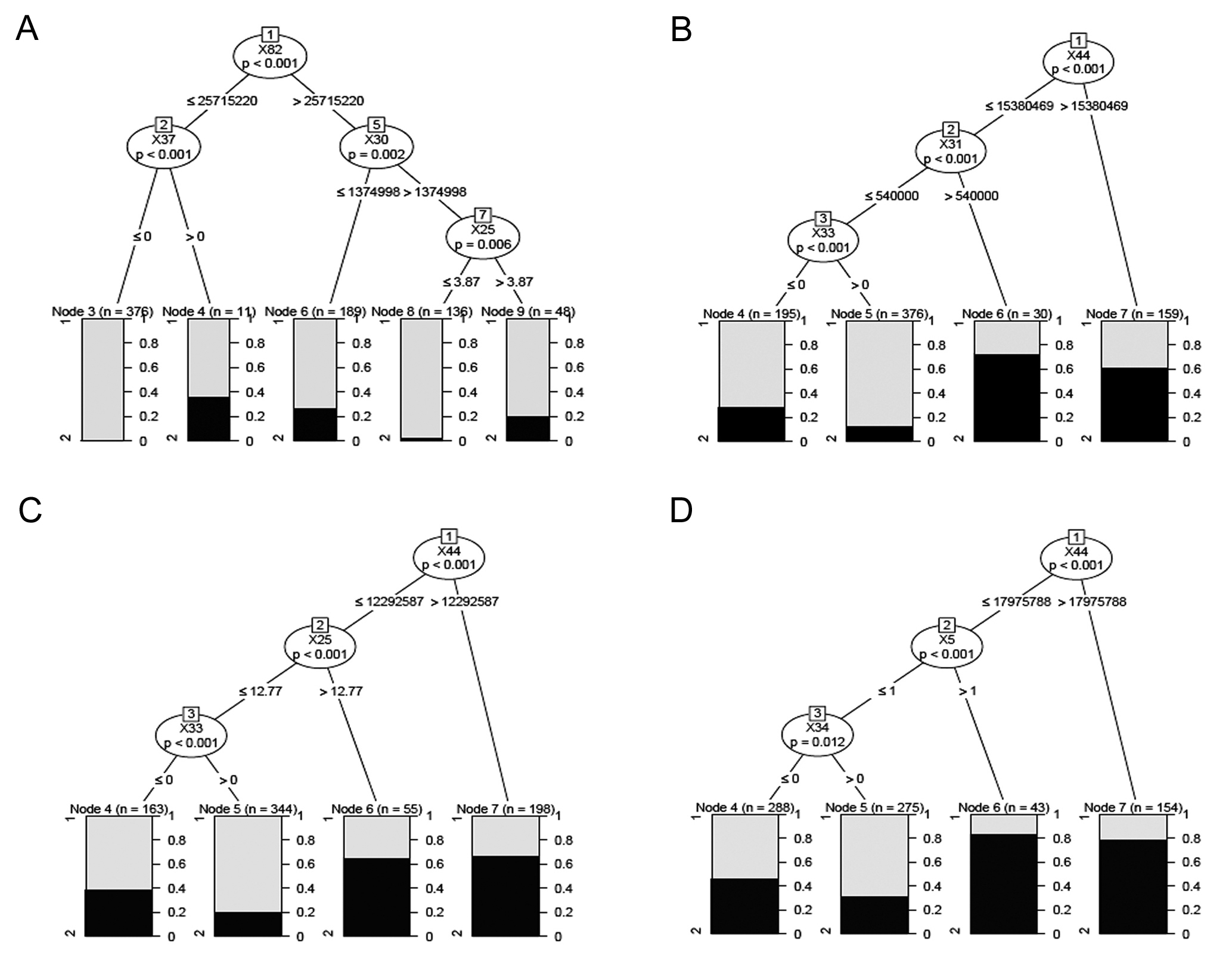

Supplement: Additional file 6 — Classification trees for downregulated genes on chromosome 17. Classification trees comparing (a) 10% most downregulated genes on chromosome 17 with all other genes. The first split divides the chromosome into two parts, proximal and distal of 35 Mb, based on the distance between the gene and a unique Lx5 element at 9.5 Mb (×82). The left side then shows that for the proximal 35 Mb of the chromosome downregulation is associated with location within a high L1 density (HL1) domain truncated by 500 kb at both ends (×37 >0). For genes distal to 35 Mb downregulation is either associated with location in a low L1 density (LL1) domain less than 1.3 Mb in size (×30), or in larger LL1 domains associated with L1 density downstream of a gene (×25) >3.7%. For trees comparing (b) 30%, (c) 40% and (d) 50% most downregulated genes on chromosome 17 with all other genes, the first split from the top node divides the chromosome into two parts, proximal and distal of approximately 70 Mb, based on the distance between the gene and the nearest full-length (FL) L1_Mus2 element (×44) which can be found at four locations on chromosome 17 (20.7, 31.6, 41.2 and 54.5 Mb). In the proximal 70 Mb of the chromosome, the models suggest that genes less susceptible to silencing are located centrally in large LL1 domains, not associated with large HL1s, and/or surrounded by a low (≤ 12.77%) local L1 density. ×5, overlaps (1) or not (0) with an HL1 domain; ×25, L1 density 100 kb upstream of the gene; ×31, size of HL1 domain with which the gene is associated; ×33 and ×34 whether a gene overlaps (1) or not (0) with a core LL1 domain (LL1 truncated by 250 kb or 500 kb, respectively, at its proximal and distal boundaries). Y-axis denotes probability of a gene being silenced (black fill). [file 1756-8935-3-10-S6.TIFF]

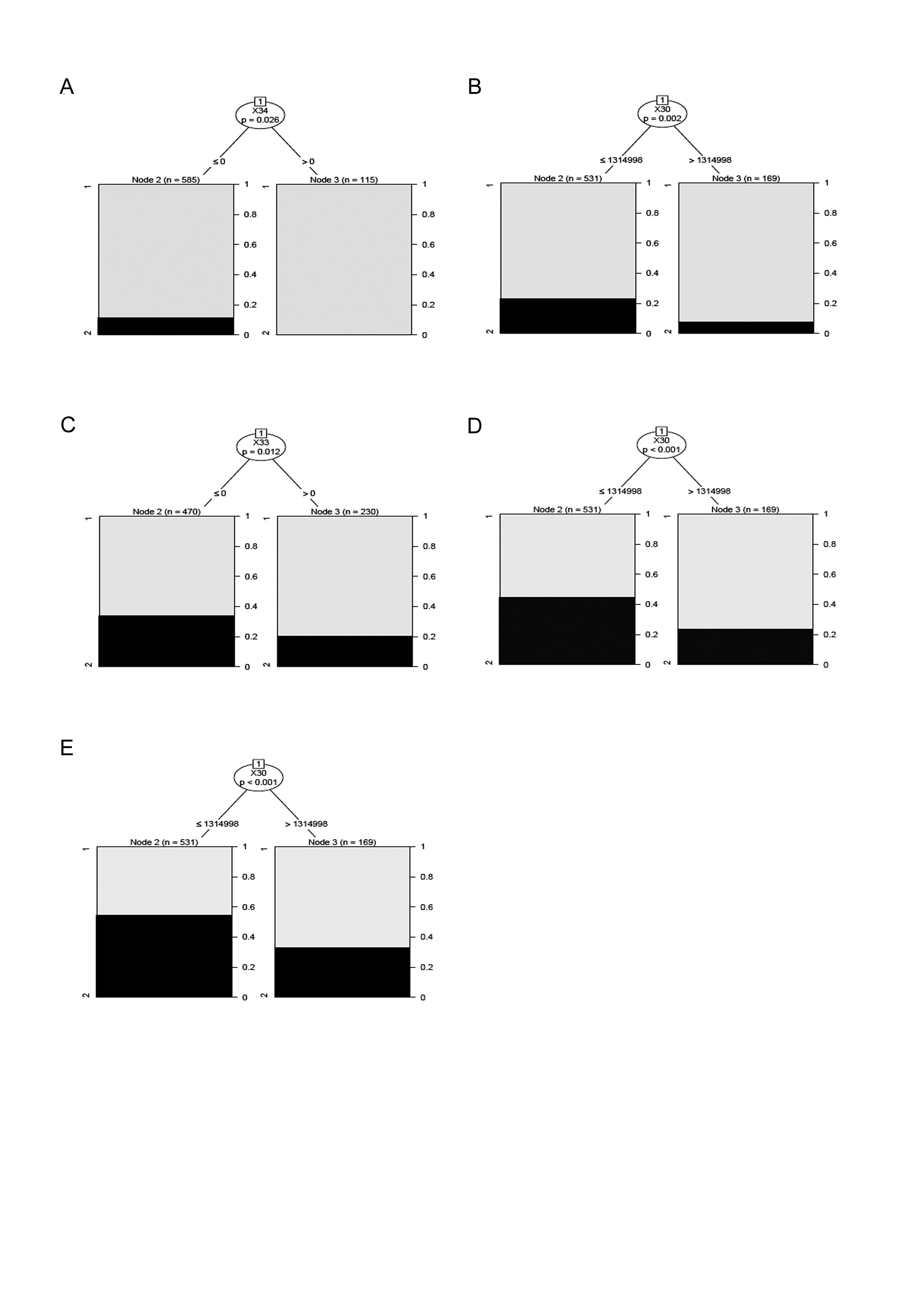

Supplement: Additional file 7 — Genes located in relatively large low L1 density (LL1) domains on chromosome 3 have a lower probability of being silenced. This effect was seen across the first five deciles, in classification trees comparing (a) 10%, (b) 20%, (c) 30%, (d) 40% and (e) 50% most downregulated genes against all other genes. For every decile, the tree estimated from the data only contained one split, which is either based on a LL1 domain size measure (×30), or on the location of the gene with respect to large LL1 domains (×33 and ×34). Generally, genes located in LL1 domains >1.31 Mb in size or those associated with 'core' LL1 regions (×33 or ×34 >0) have approximately 50% less chance of being silenced. Additional files 4 and 9 give further details on feature definition. [file 1756-8935-3-10-S7.TIFF]

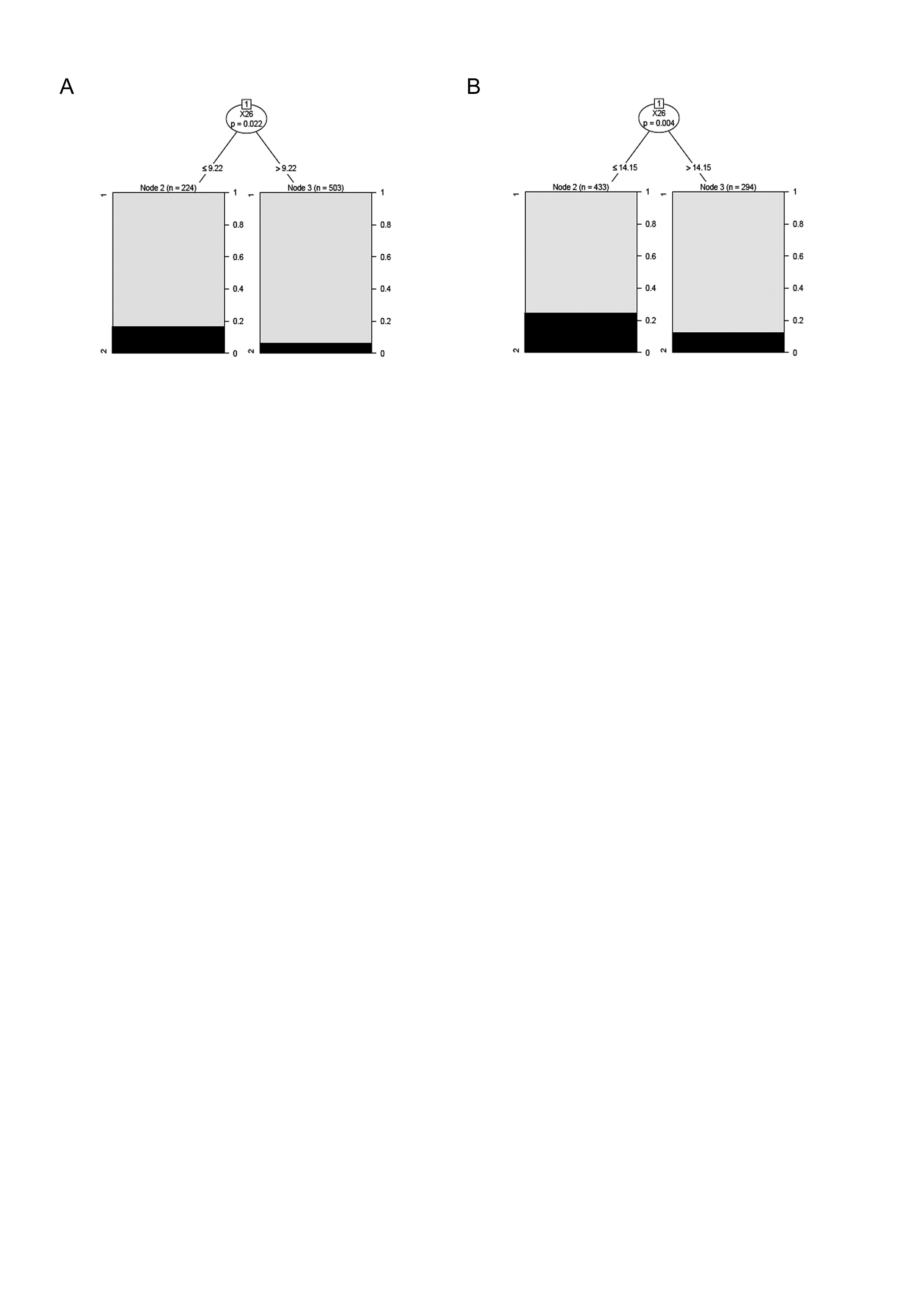

Supplement: Additional file 8 — Lower local short interspersed nuclear elements (SINE) density may be associated with gene silencing on chromosome 12. Classification trees for chromosome 12 data identified a reciprocal relationship between iXist mediated silencing and SINE density. The effect was only observed when we compared the 10% (a) and 20% (b) most strongly downregulated genes on the chromosome to all other genes. ×26, SINE density in the 100 kb region upstream of the gene. [file 1756-8935-3-10-S8.TIFF]

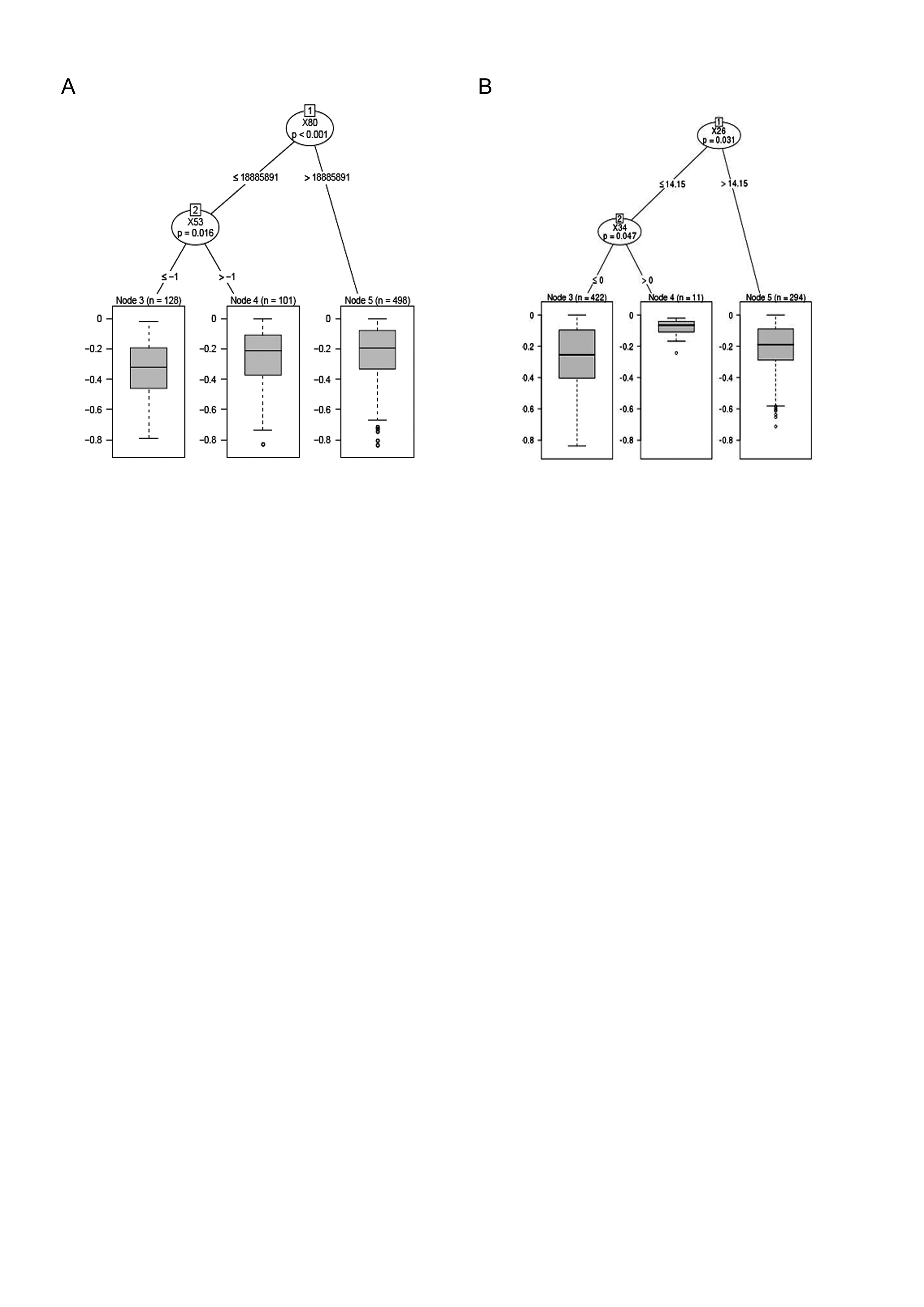

Supplement: Additional file 9 — Regression trees for chromosome 12. (a) The first split (×80) measures the distance between the gene to the nearest full length (FL) Lx4B element (which is uniquely located at approximately 6.5 Mb), followed by the second split (×53) which records the orientation of the nearest FL L1 Md_F2 to the gene (1 = upstream of the gene, -1 = downstream). (There are 35 copies of FL L1 Md_F2 on chromosome 12 altogether.) The regression tree suggests that the gene silencing effect would be the strongest for a given gene not more than 18.89 Mb away from the FL Lx4B element, (in other words, in the proximal 25.5 Mb of the chromosome), with the nearest FL L1 Md_F2 element being downstream of the gene. (b) When estimated on a subset of genomic features, the regression tree modelled the gene silencing effect differently. Here the gene silencing effect is the strongest for genes with a relatively low local SINE density (≤ 14.5%) and not associated with the core of a large low L1 density (LL1) domain (×34 = 0). Note that genes located in the centre of a large LL1 domain have a median downregulation level of just 10% (node 4 of the tree), which is a very mild effect. Additional file 4 gives details on genomic feature definition. [file 1756-8935-3-10-S9.TIFF]
