# Supplementary material for: Efficiency of Xist-mediated silencing on autosomes is linked to chromosomal domain organisation
Source: Epigenetics Chromatin. 2010 May 7;3:10. doi: 10.1186/1756-8935-3-10 (PMC2873326; doi:10.1186/1756-8935-3-10)
Supplement: Additional file 4 — Supplementary table 1. File contains a list of features used in multivariate analysis. [file 1756-8935-3-10-S4.DOC]

Table S1 List of features used in multivariate analysis

| **Features** | **Detailed name** | **Description** |
| --- | --- | --- |
| **X1** | Gene Size | Size of the gene in basepairs, calculated by (gene end position - gene start position + 1) |
| **X2** | Total intron size | A sum of sizes of all introns for the longest Ensembl transcript of the gene in basepairs. |
| **X3** | Distance from transgene | Measured in basepairs as the distance between midpoint of a gene to the approximately mapped mid-point of the transgene |
| **X4** | LL1 nesting | Whether a gene is proximally overalapping (1), distally overlapping (2), nested (3) or not overlapping at all (0) with a low-L1 (LL1) region. ("Proximal" refers to the beginning of the LL1 region.) LL1 regions were defined as the reciprocal of HL1 regions (see variable X5), excluding regions < 250kb. Centromeric regions (~3Mb) were not sequenced (always L1 low) and are removed from the LL1 list. |
| **X5** | HL1 nesting | Whether a gene is proximally overalapping (1), distally overlapping (2), nested (3) or not overlapping at all (0) with a high-L1 (HL1) region. (Proximal refers to the beginning of the HL1 region.) High L1 is defined as having at least 1 L1 element of >=5kb per 100kb, excluding regions <250kb. |
| **X6** | 10kb L1 % UP | L1 density in the 10kb region upstream of the gene |
| **X7** | 10kb L1 % DOWN | L1 density in the 10kb region downstream of the gene |
| **X8** | 10kb SINE % UP | SINE density in the 10kb region upstream of the gene |
| **X9** | 10kb SINE % DOWN | SINE density in the 10kb region downstream of the gene |
| **X10** | 10kb LTR % UP | LTR density in the 10kb region upstream of the gene |
| **X11** | 10kb LTR % DOWN | LTR density in the 10kb region downstream of the gene |
| **X12** | 25kb L1 % UP | L1 density in the 25kb region upstream of the gene |
| **X13** | 25kb L1 % DOWN | L1 density in the 25kb region downstream of the gene |
| **X14** | 25kb SINE % UP | SINE density in the 25kb region upstream of the gene |
| **X15** | 25kb SINE % DOWN | SINE density in the 25kb region downstream of the gene |
| **X16** | 25kb LTR % UP | LTR density in the 25kb region upstream of the gene |
| **X17** | 25kb LTR % DOWN | LTR density in the 25kb region downstream of the gene |
| **X18** | 50kb L1 % UP | L1 density in the 50kb region upstream of the gene |
| **X19** | 50kb L1 % DOWN | L1 density in the 50kb region downstream of the gene |
| **X20** | 50kb SINE % UP | SINE density in the 50kb region upstream of the gene |
| **X21** | 50kb SINE % DOWN | SINE density in the 50kb region downstream of the gene |
| **X22** | 50kb LTR % UP | LTR density in the 50kb region upstream of the gene |
| **X23** | 50kb LTR % DOWN | LTR density in the 50kb region downstream of the gene |
| **X24** | 100kb L1 % UP | L1 density in the 100kb region upstream of the gene |
| **X25** | 100kb L1 % DOWN | L1 density in the 100kb region downstream of the gene |
| **X26** | 100kb SINE % UP | SINE density in the 100kb region upstream of the gene |
| **X27** | 100kb SINE % DOWN | SINE density in the 100kb region downstream of the gene |
| **X28** | 100kb LTR % UP | LTR density in the 100kb region upstream of the gene |
| **X29** | 100kb LTR % DOWN | LTR density in the 100kb region downstream of the gene |
| **X30** | LL1 region size | Size of the low-L1 domain (see feature X4 above). This value is "null" if the LL1 nesting status is "0" (no overlap at all between gene and LL1 region) |
| **X31** | HL1 region size | Size of the high-L1 domain (see feature X5 above). This value is "null" if the HL1 nesting status is "0" (no overlap at all between gene and HL1 region) |
| **X32** | LL1 100kb truncated nesting | With 100kb taken away from each end of the defined LL1 region, check if a gene overlaps with the remaining region (class 1) or not (class 0) |
| **X33** | LL1 250kb truncated nesting | With 250kb taken away from each end of the defined LL1 region, check if a gene overlaps with the remaining region (class 1) or not (class 0) |
| **X34** | LL1 500kb truncated nesting | With 500kb taken away from each end of the defined LL1 region, check if a gene overlaps with the remaining region (class 1) or not (class 0) |
| **X35** | HL1 100kb truncated nesting | With 100kb taken away from each end of the defined HL1 region, check if a gene overlaps with the remaining region (class 1) or not (class 0) |
| **X36** | HL1 250kb truncated nesting | With 250kb taken away from each end of the defined HL1 region, check if a gene overlaps with the remaining region (class 1) or not (class 0) |
| **X37** | HL1 500kb truncated nesting | With 500kb taken away from each end of the defined HL1 region, check if a gene overlaps with the remaining region (class 1) or not (class 0) |
| **X38** | Distance to the nearest FL_L1 (any class) | Distance between the start or end of a gene to its nearest FL_L1 element irrespective of L1 subfamily in basepairs |
| **X39** | Orientation of the nearest FL_L1 in X38 | Orientation of the nearest FL_L1 element recorded in X38. 1 = upstream of the gene, -1 = downstream of the gene. |
| **X40** | Distance to the nearest FL L1_Mur1 | Distance between the start or end of a gene to its nearest FL L1_Mur1 in basepairs |
| **X41** | Orientation of the nearest FL L1_Mur1 | Orientation of the nearest FL_L1 element recorded in X40. 1 = upstream of the gene, -1 = downstream of the gene. |
| **X42** | Distance to the nearest FL L1_Mus1 | Distance between the start or end of a gene to its nearest FL L1_Mus1 in basepairs |
| **X43** | Orientation of the nearest FL L1_Mus1 | Orientation of the nearest FL_L1 element recorded in X42. 1 = upstream of the gene, -1 = downstream of the gene. |
| **X44** | Distance to the nearest FL L1_Mus2 | Distance between the start or end of a gene to its nearest FL L1_Mus2 in basepairs |
| **X45** | Orientation of the nearest FL L1_Mus2 | Orientation of the nearest FL_L1 element recorded in X44. 1 = upstream of the gene, -1 = downstream of the gene. |
| **X46** | Distance to the nearest FL L1_Mus3 | Distance between the start or end of a gene to its nearest FL L1_Mus3 in basepairs |
| **X47** | Orientation of the nearest FL L1_Mus3 | Orientation of the nearest FL_L1 element recorded in X46. 1 = upstream of the gene, -1 = downstream of the gene. |
| **X48** | Distance to the nearest FL L1_Mus4 | Distance between the start or end of a gene to its nearest FL L1_Mus4 in basepairs |
| **X49** | Orientation of the nearest FL L1_Mus4 | Orientation of the nearest FL_L1 element recorded in X48. 1 = upstream of the gene, -1 = downstream of the gene. |
| **X50** | Distance to the nearest FL L1Md_A | Distance between the start or end of a gene to its nearest FL L1Md_A in basepairs |
| **X51** | Orientation of the nearest FL L1Md_A | Orientation of the nearest FL_L1 element recorded in X50. 1 = upstream of the gene, -1 = downstream of the gene. |
| **X52** | Distance to the nearest FL L1Md_F2 | Distance between the start or end of a gene to its nearest FL L1Md_F2 in basepairs |
| **X53** | Orientation of the nearest FL L1Md_F2 | Orientation of the nearest FL_L1 element recorded in X52. 1 = upstream of the gene, -1 = downstream of the gene. |
| **X54** | Distance to the nearest FL L1Md_F3 | Distance between the start or end of a gene to its nearest FL L1Md_F3 in basepairs |
| **X55** | Orientation of the nearest FL L1Md_F3 | Orientation of the nearest FL_L1 element recorded in X54. 1 = upstream of the gene, -1 = downstream of the gene. |
| **X56** | Distance to the nearest FL L1Md_Gf | Distance between the start or end of a gene to its nearest FL L1Md_Gf in basepairs |
| **X57** | Orientation of the nearest FL L1Md_Gf | Orientation of the nearest FL_L1 element recorded in X56. 1 = upstream of the gene, -1 = downstream of the gene. |
| **X58** | Distance to the nearest FL L1Md_T | Distance between the start or end of a gene to its nearest FL L1Md_T in basepairs |
| **X59** | Orientation of the nearest FL L1Md_T | Orientation of the nearest FL_L1 element recorded in X58. 1 = upstream of the gene, -1 = downstream of the gene. |
| **X60** | Distance to the nearest FL L1VL4 | Distance between the start or end of a gene to its nearest FL L1VL4 in basepairs |
| **X61** | Orientation of the nearest FL L1VL4 | Orientation of the nearest FL_L1 element recorded in X60. 1 = upstream of the gene, -1 = downstream of the gene. |
| **X62** | Distance to the nearest FL L1_Mur2 | Distance between the start or end of a gene to its nearest FL L1_Mur2 in basepairs |
| **X63** | Orientation of the nearest FL L1_Mur2 | Orientation of the nearest FL_L1 element recorded in X62. 1 = upstream of the gene, -1 = downstream of the gene. |
| **X64** | Distance to the nearest FL L1_Mur3 | Distance between the start or end of a gene to its nearest FL L1_Mur3 in basepairs |
| **X65** | Orientation of the nearest FL L1_Mur3 | Orientation of the nearest FL_L1 element recorded in X64. 1 = upstream of the gene, -1 = downstream of the gene. |
| **X66** | Distance to the nearest FL L1VL1 | Distance between the start or end of a gene to its nearest FL L1VL1 in basepairs |
| **X67** | Orientation of the nearest FL L1VL1 | Orientation of the nearest FL_L1 element recorded in X66. 1 = upstream of the gene, -1 = downstream of the gene. |
| **X68** | Distance to the nearest FL L1VL2 | Distance between the start or end of a gene to its nearest FL L1VL2 in basepairs |
| **X69** | Orientation of the nearest FL L1VL2 | Orientation of the nearest FL_L1 element recorded in X68. 1 = upstream of the gene, -1 = downstream of the gene. |
| **X70** | Distance to the nearest FL L1Md_F | Distance between the start or end of a gene to its nearest FL L1Md_F in basepairs |
| **X71** | Orientation of the nearest FL L1Md_F | Orientation of the nearest FL_L1 element recorded in X70. 1 = upstream of the gene, -1 = downstream of the gene. |
| **X72** | Distance to the nearest FL Lx | Distance between the start or end of a gene to its nearest FL Lx in basepairs |
| **X73** | Orientation of the nearest FL Lx | Orientation of the nearest FL_L1 element recorded in X72. 1 = upstream of the gene, -1 = downstream of the gene. |
| **X74** | Distance to the nearest FL Lx2B | Distance between the start or end of a gene to its nearest FL Lx2B in basepairs |
| **X75** | Orientation of the nearest FL Lx2B | Orientation of the nearest FL_L1 element recorded in X74. 1 = upstream of the gene, -1 = downstream of the gene. |
| **X76** | Distance to the nearest FL Lx3_Mus | Distance between the start or end of a gene to its nearest FL Lx3_Mus basepairs |
| **X77** | Orientation of the nearest FL Lx3 | Orientation of the nearest FL_L1 element recorded in X76. 1 = upstream of the gene, -1 = downstream of the gene. |
| **X78** | Distance to the nearest FL Lx3A | Distance between the start or end of a gene to its nearest FL Lx3A in basepairs |
| **X79** | Orientation of the nearest FL Lx3A | Orientation of the nearest FL_L1 element recorded in X78. 1 = upstream of the gene, -1 = downstream of the gene. |
| **X80** | Distance to the nearest FL Lx4B | Distance between the start or end of a gene to its nearest FL Lx4B in basepairs |
| **X81** | Orientation of the nearest FL Lx4B | Orientation of the nearest FL_L1 element recorded in X80. 1 = upstream of the gene, -1 = downstream of the gene. |
| **X82** | Distance to the nearest FL Lx5 | Distance between the start or end of a gene to its nearest FL Lx5 in basepairs |
| **X83** | Orientation of the nearest FL Lx5 | Orientation of the nearest FL_L1 element recorded in X82. 1 = upstream of the gene, -1 = downstream of the gene. |
