# Supplementary material for: Efficiency of Xist-mediated silencing on autosomes is linked to chromosomal domain organisation
Source: Epigenetics Chromatin. 2010 May 7;3:10. doi: 10.1186/1756-8935-3-10 (PMC2873326; doi:10.1186/1756-8935-3-10)
Supplement: Additional file 5 — Supplementary table 2. File contains Ensembl coordinates (version 46.36 g) for the position of high L1 density (HL1) and low L1 density (LL1) domains on chromosomes 3, 12 and 17. [file 1756-8935-3-10-S5.DOC]

Table S2. HL1 and LL1 domains on chromosomes 3, 12 and 17

| **Chromosome 3** | |  |  |  |  |
| --- | --- | --- | --- | --- | --- |
|  |  |  |  |  |  |
| **HL1** |  |  |  | **LL1** |  |
|  |  |  |  |  |  |
| **start** | **end** |  |  | **start** | **end** |
| 2960000 | 3530000 |  |  | 8995001 | 9259999 |
| 3595000 | 5240000 |  |  | 9465001 | 9749999 |
| 5315000 | 8565000 |  |  | 10085001 | 10339999 |
| 8710000 | 8995000 |  |  | 19390001 | 19684999 |
| 9750000 | 10085000 |  |  | 21810001 | 22329999 |
| 10340000 | 11195000 |  |  | 25600001 | 26194999 |
| 11245000 | 13460000 |  |  | 27240001 | 28599999 |
| 13840000 | 14550000 |  |  | 30205001 | 31439999 |
| 14615000 | 15655000 |  |  | 32605001 | 33259999 |
| 15900000 | 17290000 |  |  | 34225001 | 34634999 |
| 17310000 | 19390000 |  |  | 34840001 | 35214999 |
| 20535000 | 21810000 |  |  | 38505001 | 38849999 |
| 22330000 | 23875000 |  |  | 40640001 | 41179999 |
| 23945000 | 24665000 |  |  | 50255001 | 50534999 |
| 25035000 | 25600000 |  |  | 51150001 | 52414999 |
| 26480000 | 26740000 |  |  | 54315001 | 55184999 |
| 26790000 | 27240000 |  |  | 67980001 | 68504999 |
| 28955000 | 30205000 |  |  | 68710001 | 69899999 |
| 31440000 | 32070000 |  |  | 81965001 | 83069999 |
| 32250000 | 32605000 |  |  | 83525001 | 84744999 |
| 33260000 | 33540000 |  |  | 85270001 | 86139999 |
| 33550000 | 34225000 |  |  | 86520001 | 87294999 |
| 35215000 | 35670000 |  |  | 87895001 | 90699999 |
| 36790000 | 37385000 |  |  | 92205001 | 92529999 |
| 38035000 | 38505000 |  |  | 93160001 | 93869999 |
| 39115000 | 40150000 |  |  | 94915001 | 96844999 |
| 40195000 | 40640000 |  |  | 97630001 | 98379999 |
| 41180000 | 41535000 |  |  | 100600001 | 101914999 |
| 41760000 | 42930000 |  |  | 102120001 | 102419999 |
| 42955000 | 43715000 |  |  | 103215001 | 103529999 |
| 43805000 | 45550000 |  |  | 103740001 | 104144999 |
| 45885000 | 46665000 |  |  | 104425001 | 105269999 |
| 46730000 | 47270000 |  |  | 105570001 | 106074999 |
| 47275000 | 49675000 |  |  | 107155001 | 108014999 |
| 49790000 | 50255000 |  |  | 108220001 | 108969999 |
| 50535000 | 50895000 |  |  | 115535001 | 115804999 |
| 52790000 | 53105000 |  |  | 116010001 | 116439999 |
| 53625000 | 54030000 |  |  | 116645001 | 116919999 |
| 55860000 | 56790000 |  |  | 117625001 | 117994999 |
| 56835000 | 57545000 |  |  | 120200001 | 120514999 |
| 57990000 | 59045000 |  |  | 121145001 | 123084999 |
| 59265000 | 59875000 |  |  | 123290001 | 123619999 |
| 60220000 | 60635000 |  |  | 126155001 | 126784999 |
| 61065000 | 61400000 |  |  | 127235001 | 127659999 |
| 61430000 | 62475000 |  |  | 127870001 | 128329999 |
| 62500000 | 63860000 |  |  | 129120001 | 130394999 |
| 64225000 | 65615000 |  |  | 130865001 | 131539999 |
| 66055000 | 66465000 |  |  | 134020001 | 134284999 |
| 66615000 | 67105000 |  |  | 135200001 | 135639999 |
| 67235000 | 67725000 |  |  | 135845001 | 136344999 |
| 69900000 | 70545000 |  |  | 136550001 | 136964999 |
| 70660000 | 71635000 |  |  | 137710001 | 137999999 |
| 71675000 | 71935000 |  |  | 138430001 | 139014999 |
| 72010000 | 72465000 |  |  | 142045001 | 142314999 |
| 72495000 | 73210000 |  |  | 143065001 | 143674999 |
| 73285000 | 73795000 |  |  | 144070001 | 144489999 |
| 74195000 | 75230000 |  |  | 145095001 | 145699999 |
| 75300000 | 75650000 |  |  | 146395001 | 146939999 |
| 75765000 | 76030000 |  |  | 147395001 | 147979999 |
| 76070000 | 76440000 |  |  | 148280001 | 148539999 |
| 76455000 | 76945000 |  |  | 148745001 | 149484999 |
| 77135000 | 77565000 |  |  | 150220001 | 150554999 |
| 77650000 | 78000000 |  |  | 152000001 | 152404999 |
| 78315000 | 79160000 |  |  | 152610001 | 153859999 |
| 79620000 | 81965000 |  |  | 154065001 | 154329999 |
| 83070000 | 83525000 |  |  |  |  |
| 86140000 | 86520000 |  |  |  |  |
| 87295000 | 87660000 |  |  |  |  |
| 90700000 | 91355000 |  |  |  |  |
| 91385000 | 92205000 |  |  |  |  |
| 92530000 | 92890000 |  |  |  |  |
| 93870000 | 94475000 |  |  |  |  |
| 94530000 | 94915000 |  |  |  |  |
| 98380000 | 99235000 |  |  |  |  |
| 99385000 | 99845000 |  |  |  |  |
| 102420000 | 102670000 |  |  |  |  |
| 102840000 | 103215000 |  |  |  |  |
| 104145000 | 104425000 |  |  |  |  |
| 105270000 | 105570000 |  |  |  |  |
| 106075000 | 107155000 |  |  |  |  |
| 108970000 | 109490000 |  |  |  |  |
| 110015000 | 110675000 |  |  |  |  |
| 111585000 | 114075000 |  |  |  |  |
| 114120000 | 114370000 |  |  |  |  |
| 114385000 | 115070000 |  |  |  |  |
| 115175000 | 115535000 |  |  |  |  |
| 117995000 | 118410000 |  |  |  |  |
| 119865000 | 120200000 |  |  |  |  |
| 120515000 | 121145000 |  |  |  |  |
| 123620000 | 124575000 |  |  |  |  |
| 124720000 | 125470000 |  |  |  |  |
| 125660000 | 126155000 |  |  |  |  |
| 128580000 | 129120000 |  |  |  |  |
| 131540000 | 131985000 |  |  |  |  |
| 132135000 | 132640000 |  |  |  |  |
| 132660000 | 134020000 |  |  |  |  |
| 134285000 | 135200000 |  |  |  |  |
| 137240000 | 137710000 |  |  |  |  |
| 139015000 | 139605000 |  |  |  |  |
| 140560000 | 141095000 |  |  |  |  |
| 141440000 | 141800000 |  |  |  |  |
| 142315000 | 143065000 |  |  |  |  |
| 143675000 | 144070000 |  |  |  |  |
| 144490000 | 145095000 |  |  |  |  |
| 146130000 | 146395000 |  |  |  |  |
| 146940000 | 147395000 |  |  |  |  |
| 147980000 | 148280000 |  |  |  |  |
| 149915000 | 150220000 |  |  |  |  |
| 150940000 | 151210000 |  |  |  |  |
| 151460000 | 151725000 |  |  |  |  |
| 154330000 | 154680000 |  |  |  |  |
| 154735000 | 155370000 |  |  |  |  |
| 156120000 | 156475000 |  |  |  |  |
| 156795000 | 157045000 |  |  |  |  |
| 157095000 | 157450000 |  |  |  |  |
| 157930000 | 158480000 |  |  |  |  |
| 158530000 | 159425000 |  |  |  |  |
| 159465000 | 159805000 |  |  |  |  |

| **Chromosome 12** |  |  |  |  |  |
| --- | --- | --- | --- | --- | --- |
|  |  |  |  |  |  |
| **HL1** |  |  |  | **LL1** |  |
|  |  |  |  |  |  |
| **start** | **end** |  |  | **start** | **end** |
| 5755000 | 6995000 |  |  | 3205001 | 3514999 |
| 7035000 | 7310000 |  |  | 3725001 | 5404999 |
| 7725000 | 8320000 |  |  | 8320001 | 9109999 |
| 9550000 | 9805000 |  |  | 9805001 | 10549999 |
| 10550000 | 11315000 |  |  | 12595001 | 13344999 |
| 11415000 | 11830000 |  |  | 15995001 | 16334999 |
| 11880000 | 12315000 |  |  | 16750001 | 17419999 |
| 13345000 | 14175000 |  |  | 18950001 | 19504999 |
| 14310000 | 15750000 |  |  | 19975001 | 20834999 |
| 18135000 | 18520000 |  |  | 21415001 | 21734999 |
| 20835000 | 21135000 |  |  | 23400001 | 23779999 |
| 21735000 | 21985000 |  |  | 24155001 | 24644999 |
| 22165000 | 22540000 |  |  | 25130001 | 26229999 |
| 22620000 | 22915000 |  |  | 26740001 | 27394999 |
| 23780000 | 24155000 |  |  | 28880001 | 29864999 |
| 27395000 | 27645000 |  |  | 30190001 | 30569999 |
| 28175000 | 28880000 |  |  | 30875001 | 31149999 |
| 29865000 | 30190000 |  |  | 31795001 | 32739999 |
| 30570000 | 30875000 |  |  | 33435001 | 33944999 |
| 31150000 | 31795000 |  |  | 34950001 | 35214999 |
| 32740000 | 33435000 |  |  | 35420001 | 35829999 |
| 33945000 | 34355000 |  |  | 37100001 | 37359999 |
| 34360000 | 34950000 |  |  | 40985001 | 41544999 |
| 35830000 | 36130000 |  |  | 41990001 | 42849999 |
| 36685000 | 37100000 |  |  | 46390001 | 46809999 |
| 37360000 | 38350000 |  |  | 47445001 | 47799999 |
| 38370000 | 39185000 |  |  | 50835001 | 51174999 |
| 39320000 | 39620000 |  |  | 52195001 | 52929999 |
| 39770000 | 40150000 |  |  | 53675001 | 54029999 |
| 40305000 | 40575000 |  |  | 54235001 | 56449999 |
| 42850000 | 43750000 |  |  | 56655001 | 57019999 |
| 43805000 | 44320000 |  |  | 57600001 | 57859999 |
| 44425000 | 45230000 |  |  | 58065001 | 58414999 |
| 45250000 | 45765000 |  |  | 69950001 | 71294999 |
| 47050000 | 47445000 |  |  | 71500001 | 72024999 |
| 47800000 | 48335000 |  |  | 72610001 | 72979999 |
| 48600000 | 50835000 |  |  | 73610001 | 74234999 |
| 51505000 | 51930000 |  |  | 74440001 | 74844999 |
| 52930000 | 53240000 |  |  | 76405001 | 77974999 |
| 58415000 | 59885000 |  |  | 79640001 | 80209999 |
| 60085000 | 60620000 |  |  | 80420001 | 82169999 |
| 60765000 | 61040000 |  |  | 82570001 | 83449999 |
| 61090000 | 61465000 |  |  | 83805001 | 88284999 |
| 61870000 | 62395000 |  |  | 89285001 | 90009999 |
| 62450000 | 63330000 |  |  | 91980001 | 92239999 |
| 63640000 | 64485000 |  |  | 98620001 | 99224999 |
| 64505000 | 65095000 |  |  | 99645001 | 101094999 |
| 65115000 | 65910000 |  |  | 102910001 | 103759999 |
| 66075000 | 66375000 |  |  | 104705001 | 108914999 |
| 66730000 | 67420000 |  |  | 109120001 | 109474999 |
| 67600000 | 67945000 |  |  | 110340001 | 112399999 |
| 68095000 | 68580000 |  |  | 112605001 | 113839999 |
| 68645000 | 69090000 |  |  | 116590001 | 116844999 |
| 69190000 | 69465000 |  |  | 117610001 | 118474999 |
| 69510000 | 69950000 |  |  |  |  |
| 75675000 | 75950000 |  |  |  |  |
| 76010000 | 76405000 |  |  |  |  |
| 78200000 | 78570000 |  |  |  |  |
| 78585000 | 78900000 |  |  |  |  |
| 79120000 | 79640000 |  |  |  |  |
| 82170000 | 82570000 |  |  |  |  |
| 83450000 | 83805000 |  |  |  |  |
| 88285000 | 88845000 |  |  |  |  |
| 90990000 | 91980000 |  |  |  |  |
| 92240000 | 92620000 |  |  |  |  |
| 92895000 | 93825000 |  |  |  |  |
| 93860000 | 94345000 |  |  |  |  |
| 94640000 | 95055000 |  |  |  |  |
| 95375000 | 95840000 |  |  |  |  |
| 95875000 | 97540000 |  |  |  |  |
| 97675000 | 98325000 |  |  |  |  |
| 101435000 | 102235000 |  |  |  |  |
| 103760000 | 104380000 |  |  |  |  |
| 109475000 | 109890000 |  |  |  |  |
| 114195000 | 114895000 |  |  |  |  |
| 114935000 | 115440000 |  |  |  |  |
| 115445000 | 116590000 |  |  |  |  |
| 116845000 | 117395000 |  |  |  |  |
| 118475000 | 118930000 |  |  |  |  |
| 119315000 | 120535000 |  |  |  |  |
|  |  |  |  |  |  |

| **Chromosome 17** | |  |  |  |  |
| --- | --- | --- | --- | --- | --- |
|  |  |  |  |  |  |
| **HL1** |  |  |  | **LL1** |  |
|  |  |  |  |  |  |
| **start** | **end** |  |  | **start** | **end** |
| 3480000 | 4140000 |  |  | 4955001 | 9079999 |
| 4435000 | 4955000 |  |  | 9750001 | 10389999 |
| 9080000 | 9510000 |  |  | 11960001 | 12294999 |
| 10690000 | 10945000 |  |  | 12500001 | 12824999 |
| 11035000 | 11440000 |  |  | 13030001 | 13469999 |
| 13470000 | 13920000 |  |  | 13920001 | 14194999 |
| 14195000 | 14735000 |  |  | 14735001 | 15724999 |
| 15725000 | 17020000 |  |  | 18965001 | 19269999 |
| 17155000 | 17490000 |  |  | 23280001 | 29794999 |
| 17565000 | 18965000 |  |  | 30330001 | 31509999 |
| 19270000 | 19590000 |  |  | 31715001 | 32199999 |
| 19625000 | 20200000 |  |  | 32430001 | 32759999 |
| 20240000 | 20665000 |  |  | 32990001 | 34029999 |
| 20675000 | 23280000 |  |  | 34475001 | 35849999 |
| 35850000 | 36455000 |  |  | 36455001 | 36794999 |
| 36795000 | 37565000 |  |  | 42005001 | 42544999 |
| 37690000 | 40615000 |  |  | 42750001 | 43299999 |
| 41050000 | 41775000 |  |  | 43675001 | 44274999 |
| 43300000 | 43675000 |  |  | 44765001 | 50064999 |
| 44275000 | 44765000 |  |  | 50495001 | 51079999 |
| 51450000 | 52300000 |  |  | 55530001 | 56949999 |
| 52345000 | 52945000 |  |  | 60875001 | 61339999 |
| 53100000 | 54225000 |  |  | 62185001 | 63134999 |
| 54245000 | 55530000 |  |  | 64420001 | 64879999 |
| 56950000 | 58545000 |  |  | 65470001 | 66454999 |
| 58900000 | 59365000 |  |  | 66660001 | 66944999 |
| 59725000 | 60350000 |  |  | 67155001 | 67894999 |
| 61340000 | 61735000 |  |  | 68540001 | 69124999 |
| 63135000 | 63455000 |  |  | 70330001 | 71124999 |
| 63685000 | 64420000 |  |  | 71330001 | 72429999 |
| 64880000 | 65470000 |  |  | 72640001 | 72979999 |
| 68155000 | 68540000 |  |  | 73450001 | 73909999 |
| 69125000 | 69825000 |  |  | 74135001 | 74624999 |
| 69855000 | 70330000 |  |  | 74900001 | 75199999 |
| 74625000 | 74900000 |  |  | 77840001 | 78289999 |
| 75470000 | 76400000 |  |  | 79655001 | 80329999 |
| 77035000 | 77310000 |  |  | 80600001 | 81044999 |
| 77345000 | 77840000 |  |  | 83425001 | 88259999 |
| 78850000 | 79135000 |  |  | 90035001 | 90629999 |
| 80330000 | 80600000 |  |  |  |  |
| 81045000 | 81360000 |  |  |  |  |
| 81490000 | 82190000 |  |  |  |  |
| 82195000 | 82990000 |  |  |  |  |
| 88485000 | 88740000 |  |  |  |  |
| 88835000 | 90035000 |  |  |  |  |
| 91055000 | 92240000 |  |  |  |  |
| 92325000 | 92945000 |  |  |  |  |
| 93080000 | 93540000 |  |  |  |  |
| 93695000 | 94055000 |  |  |  |  |
| 94220000 | 95195000 |  |  |  |  |
|  |  |  |  |  |  |
