# Supplementary material for: Efficiency of Xist-mediated silencing on autosomes is linked to chromosomal domain organisation
Source: Epigenetics Chromatin. 2010 May 7;3:10. doi: 10.1186/1756-8935-3-10 (PMC2873326; doi:10.1186/1756-8935-3-10)
Supplement: Additional file 10 — Supplementary methods. File includes supplementary methods for RNA and DNA fluorescence in situ hybridisation (FISH) analysis, immunofluorescence, detailed description of statistical analysis, genomic data analysis and supplementary references. [file 1756-8935-3-10-S10.PDF]

Supplementary methods

DNA FISH analysis

Metaphase spreads were prepared as described elsewhere [1]. DNA FISH was performed essentially as described [2], except for omitting the 0.7% Triton X-100/HCl incubation step. Digoxygenin-11-dUTP or biotin-16-dUTP-(Roche) labelled plasmid DNA was used as specific probes. Transgenic Xist was detected with pTREXist and endogenous Xist was detected with a BAC clone covering ~100kb of genomic DNA surrounding and enclosing the *Xist* locus. An X-chromosome-specific probe DXSmh141 derived from a block of tandem repeats in band A3 was used to mark the X-chromosome [3]. Probes derived from BACs containing chromosome-specific pericentromeric repeats [4] were used to identify the chromosome on which the transgene has integrated.

The biotinylated probes were detected with avidin-texas red followed by biotinylated anti-avidin and a final layer of avidin-texas red. DIG-labelled probes were detected with anti-DIG FITC (Roche) followed by a second layer of anti-sheep FITC. All antibody were from Vector Laboratories unless otherwise stated. Images were captured on a Leica DMRB fluorescence microscope by using a Photometrics charge-coupled device camera and QUIPS software (Applied Imaging, San Jose, CA).

RNA FISH analysis

RNA FISH was performed essentially as described [5, 6]. pGPT16 (*Xist* exon 1) or pBSXist plasmid was used to detect *Xist* RNA. Labelling and detection of the probe was as described above. Xist RNA FISH images on metaphase

spreads were acquired on a Leica TCS SP5 confocal microscope using LAS AF software.

Immunofluorescence

H3K27me3/uH2A double immunofluorescence was carried out as described previously [7]. Cells were cytospun onto slides in PBS, permeabilised for 5 min in 0.4% Triton X-100 and fixed for 15 min in 2% paraformaldehyde solution followed by incubation with specific antibody. Images were collected on a Leica TCS SP5 confocal microscope using LAS AF software.

Statistical analysis

Differential expression analysis

Prior to microarray data preprocessing, it was confirmed that the quality of all microarray data satisfied both the Affymetrix recommended and CSC/IC Microarray Centre in-house quality control criteria. Processing of microarray data was performed using Bioconductor packages in R.

For each cell line, 25-mer probe hybridisation intensity data from all arrays (twelve for cell lines 8A and 3E, ten for cell line 12B) were preprocessed using the Robust Multichip Average (RMA) algorithm in the "affy" R package [8]. The arrays from one experiment were preprocessed together as they showed a similar data structure, judging from boxplots of perfect-match probe hybridisation intensities. The RMA algorithm was chosen as it provides more precise measurements for probes with lower expression values and eventually leads to higher sensitivity and specificity in detecting differential gene

expression. These strengths of the RMA algorithm are especially important in the context of this study as the maximum theoretical level of gene inactivation is only 50%. After preprocessing, probe set-based expression data have been log2-transformed. For cell lines 8A and 3E, the data sets were filtered to remove probe sets which were not expressed in any of the samples. The filtering threshold was estimated per cell line by taking the median expression data of ~100 probe sets which were known to be naturally *not* expressed in ES cells from previous experimental work (e.g. real-time PCR analyses). These "unexpressed" probe sets all map to tissue-specific genes, for example neural markers *Olig2*, *Nkx2-1*, *Dlx2* [9] immuno markers *Il3*, *Gata1*, *Tal1* [10] and extra-embryonic endoderm lineage-specific genes *Foxd3*, *Hnf4*, *Fgf5, Afp* (T. Nesterova, unpublished). The estimated threshold for filtering was 3.0841 (cell line 8A) and 4.7014 (cell line 3E), removing 38.74% and 34.21% of probe sets respectively. The chr12 data set was not filtered after RMA preprocessing because all samples were from differentiated ES cells and hence no ES microarray expression data were available for estimation of filtering threshold.

For preprocessed probe sets which have passed the filtering threshold (if required), their fold-change in expression (still log2-transformed) was calculated by subtracting the mean of control samples from the mean of treated samples. Detection of differential expression was carried out by using linear models and specifically the empirical Bayes methods [11] implemented in the R/Bioconductor package "limma". Multiple-testing correction was carried out in order to control the false discovery rate (FDR) using the methods of

Benjamini and Hochberg as implemented in the R/Bioconductor package "multtest".

To map probe sets to the genome, Ensembl microarray probes mapping data was used. Any probe set which was mapped to more than one gene was considered to be nonspecific and was discarded from analysis. In this project, 26413 out of 45037 probe sets (58.65%) were mapped to unique locations in the genome, corresponding to 16527 genes (68.66%) in the genome.

Combining differential expression and probe set mapping data, differentially expressed probe sets were defined as those which were mapped to unique locations in the genome and with a FDR of ≤5%. Out of all probe sets showing an FDR ≤5%, downregulated and upregulated ones are defined as those with an "actual" (i.e. non log-transformed) change of expression <1 and >1 respectively. E.g. a probe set with an actual change of 0.75 is 25% downregulated in the +DOX (treated) samples relative to -DOX controls. Following the definition of downregulated probe sets, downregulated genes are defined as those represented by at least one downregulated probe set and no upregulated probe set at FDR ≤5%. The expression value of a downregulated gene was calculated as the average of all associated downregulated probe sets. Genes which were not associated with any probe sets with statistically significant change were considered to be showing no change in expression.

Feature selection using CART

Further statistical modelling was performed on the experimental data with the objective to determine whether there was a specific subset of genomic features that explained the observed patterns of variation in gene expression. We employed non-parametric methods to model the conditional distribution of gene expression, given the genomic features. At the same time, we also wanted to determine the relative importance of each feature and how the salient features interact with each other. Recursive partitioning or CART (Classification and Regression Trees) models were fit to the experimental data because of their ability to model complex relationships between features, possibly measured on different scales, and deliver compact statistical representations or rules that can be easily visualized and interpreted.

For each gene, we observed a feature vector $\mathbf{x} = \{x_1, x_2, \ldots, x_p\}$, taken from a feature space $\Omega = \Omega_1 \times \Omega_2 \times \cdots \times \Omega_p$ where each x_j was one of $p = 83$ features describing a property of the gene, for instance its intrinsic characteristic (gene size, intron size), its distance from a transgene integration site, the local density of repetitive elements (L1, SINE or LTR) at various distances up/downstream of the gene, its positioning with respect to defined HL1 or LL1 domains, and the to its nearest FL-L1 element of different subfamilies -- see Supplementary Table 1 for the full list of all the observed features. The underlying assumption was that some of these features best explain the conditional distribution of gene expression fold change (observed on a $\log_2$ scale) in the two experimental conditions (+DOX and -DOX). The fold change

was used to define the response variable y being modeled non-parametrically.

Both classification and regression trees were inferred from the data. In the classification analysis, the fold changes were initially grouped into two disjoint classes determining the severity of down-regulation. Grouping fold changes into disjoint classes has the effect of attenuating the noise inherent to microarray measurements. The binary class label $y \in \{1, 2\}$ was used as response variable in the tree-based model, where the first class indicates the highest degree of down-regulation. The classification model then provides a function $d(\mathbf{x})$ defined on $\mathbf{x}$ so that for every $\mathbf{x}$, $d(\mathbf{x})$ is equal to either 1 or 2. The empirical deciles of the fold change distribution were used to determine the cutoff value that identifies the two classes. Since grouping fold changes into classes requires a somewhat arbitrary selection of cutoff values, which may not capture well the true degrees of downregulation, five different classification trees were fit to the data using the top five deciles of the fold change distribution. In the regression analysis, the continuous fold change measurement was taken as the response variable y and was modeled directly. In this case, $d(\mathbf{x})$ is a real function.

In both classification and regression cases, the conditional distribution $p(y \mid \mathbf{x})$ is approximated using a random sample of m independent and identically distributed observations. Tree-based methods learn this conditional distribution by partitioning the feature space into a number of non-overlapping

cells or regions. Each region is assigned a class (in the classification setting) or a constant value (in the regression setting). The starting point of a tree model is the *root note* which consists of the entire data set, and sits at the top of the tree. A *node* is a subset of the entire set of available features and it can be either terminal or non-terminal. A *non-terminal node* is a node that splits into two daughter nodes. Such a binary split is determined by a Boolean condition on the value of a single feature, where the condition is either satisfied or not satisfied. The sample size in each terminal node is indicated in the estimated trees.

Common fitting algorithms for CART models generally operate in two stages: all the observations are first partitioned by univariate splits in a recursive way and then a constant model is fit in each cell of the resulting partition. Usually an exhaustive search over all possible splits that maximize some measure of node impurity is performed in order to detect the features showing the best split. However, the maximization of a splitting criterion over all possible splits make the feature selection process biased, especially when the features are measured on different scales and may have missing values. *Tree pruning* strategies, generally based on cross validation, are commonly employed to select the tree having the right size and performing well in a predictive sense, thus avoiding over-fitting [12]. However, the issue of selection bias introduced during the feature selection problem still remains and is acerbated by the presence of features having a varying number of possible splits, as well as the presence of missing values.

In our setting, the purpose of the statistical modeling was to gain an understanding of what features and feature interactions drive the phenomenon of downregulation, and give simple characterizations of the conditions that determine when a gene is more severely down-regulated. To understand what features are more relevant to explain this phenomenon, it is particularly important to apply a model fitting algorithm that selects salient features of the genes in an unbiased way and discards background noise. Motivated by these arguments, we embraced the *conditional inference framework* for fitting tree-based models based on permutation tests [13, 14]. Unlike more traditional model fitting procedures, this approach explicitly deals with the problem of selection bias in feature selection by incorporating permutation-based hypothesis testing procedures for feature selection in the model building process.

In order to identify the observations contributing to each node, a weight vector $\mathbf{w} = (w_1, w_2, \ldots, w_m)$ is introduced. For a given node, each weight w_i is a binary value, either one or zero, depending on whether observation i contributes or not to the node, respectively. The suggested model fitting algorithm operates in two consecutive stages: an initial feature selection step and a subsequent splitting procedure that determines the optimal binary split on the selected feature. In the first stage, for a given weight vector $\mathbf{w}$, a global null hypothesis of no association between any of the p features and the response y is tested, and the algorithm is stopped if the data suggest that the global null hypothesis cannot be rejected. The global hypothesis of independence is given by

$$H_0 = \bigcap_{j=1}^{p} H_{0,j}$$

where each individual null hypothesis $H_{0,j}$, with $j = 1, 2, \ldots, p$, states that the response y does not depend on the feature being tested, that is $p(y \mid x_j) = p(y)$. When the global null hypothesis is rejected at a given significance level α the feature x_j having the strongest association to y (that is, smallest p-value) is selected. The association between the response and the feature vector is measured by a linear test statistic $\mathbf{T}_j$ that, in its most general form, can be written as

$$\mathbf{T}_j = \mathrm{vec}\left(\sum_{i=1}^{m} g_j(x_{ij}) h(y_i, (y_1, y_2, \ldots, y_m))^T \right)$$

where 'vec' is the operator that converts a matrix into a vector, $g_j(\bullet)$ is a transformation of feature x_{ij} and $h(\bullet)$ depends on the responses in a permutation symmetric way. In the regression analysis, we set $h(y_i, (y_1, y_2, \ldots, y_m)) = y_i$ whereas in the classification analysis $h(y_i, (y_1, y_2, \ldots, y_m)) = e_J(y_i)$ where $e_J(k)$ denotes the unit vector of length J with the *j th* element being equal to one. We have only considered two-class classification settings where $J = 2$. For features describing continuous measurements we set $g_{ij}(x) = x$ whereas for nominal features taking a value k on a set $(1, 2, \ldots, K)$ we set $g_{ij}(k) = e_K(k)$. The sampling distribution of the test statistics, which depends on the joint probability distribution $p(y, \mathbf{x})$, is not available analytically but under the null hypothesis of no dependence it can be

approximated by using permutation tests. The conditional expectations $\mathrm{E}(\mathbf{T}_j)$ and $\mathrm{Var}(\mathbf{T}_j)$ of the test statistic under the null, conditional upon all permutations of the response, are available in closed form [13] and the statistic $\mathbf{T}_j$ can be standardized. For each feature x_j, the conditional test statistic under $H_{o,j}$ is then computed and a p-value obtained that expresses the strength of association between x_j and the response. The advantage of this formulation is that p-values obtained from features measured at different scales (e.g. continuous versus nominal) can be directly compared. The p-value of each non-terminal node is reported in each one of the estimated trees.

In the second stage, the algorithm determines the best splitting value for x_j. If the optimal splitting point is denoted by $x_j^{(o)}$, the two daughter notes are determined by splitting the observations into two groups. These daughter notes are associated with left and right weight vectors, respectively $w_i^{(l)} = w_i I(x_j \leq x^{(o)})$ and $w_i^{(r)} = w_i I(x_j > x^{(o)})$, for all $i = 1, 2, \ldots, m$, where $I(\bullet)$ is the indicator function. The permutation test approach is also used in this stage in order to find the optimal binary split $x_j^{(o)}$. The algorithm then repeats the two stages on each newly created node, until no more daughter nodes are created. This results into a partition of the feature space Ω into cells, and in a tree-based representation of the model. In this respect, the significance level α, which was set to 0.05, also controls the size of the estimated tree.

Genomic data analysis

Mouse genome sequence, gene set, repetitive features and probe set mapping data were extracted from Ensembl version 46.36g (http://aug2007.archive.ensembl.org/index.html) for most analyses using custom Perl scripts implementing the Ensembl Perl API. For analyses of gene clusters (e.g. olfactory receptors, histones), Ensembl data version 40.36a was used. All genomic data were based on NCBI mouse assembly build 36.

For LINE-1 data displayed in Genome Environment Browser (GEB), L1 repeat features extracted from Ensembl were further processed by custom Perl scripts in order to display the structure of each repeat: 5' UTR, ORF1, ORF2 and 3' UTR. Due to the rapid evolution and hence large variation in L1 repeats within the UTRs (and to a lesser degree ORF1), with only ORF2 being evolutionarily conserved, RepeatMasker (Smit, AFA, Hubley, R and Green, P. *RepeatMasker Open-3.0*,1996-2004, http://www.repeatmasker.org) uses a library of L1 constituents (ORF and UTR sequences) instead of a consensus sequence for a full-length L1 element for efficient repeat prediction. In order to assemble the predicted fragments, RepeatMasker normalises position numbers relative to the conserved ORF2 in a complete L1PA2 element. The GEB predictions of the ORF and UTR positions were calculated relative to this positioning. The predicted region size of the L1 repeat can be different to the actual predicted region size on the chromosome so GEB predicted ORF and UTR sizes are normalised using the difference ratio. L1 elements which are ≥6kb with no internal inversions were annotated as full-length.

The density of repetitive elements in a region was calculated as the ratio between the size of the elements and the size of the analysed region. High-L1 (HL1) regions were defined as regions ≥250kb and having at least one L1 element ≥5kb per 100kb, identified by custom Perl scripts which scan each chromosome in 100kb sliding segments (5kb/slice). Low-L1 (LL1) regions were defined as the reciprocal of HL1 regions, again excluding regions shorter than 250kb and centromeric regions.

For the analysis of L1 density in gene clusters, the clusters were mapped to Ensembl genes with reference to published data [15, 16]. Genes must be within 500kb of each other to be classified in the same cluster, and each cluster was defined by at least three genes. The exact boundaries of each cluster were defined by the beginning of exon 1 of the first gene and the end of the last exon of the last gene.

References

1. Nesterova TB, Duthie SM, Mazurok NA, Isaenko AA, Rubtsova NV, Zakian SM, Brockdorff N: **Comparative mapping of X chromosomes in vole species of the genus Microtus.** *Chromosome Res* 1998, **6:**41-48.
2. Duthie SM, Nesterova TB, Formstone EJ, Keohane AM, Turner BM, Zakian SM, Brockdorff N: **Xist RNA exhibits a banded localization on the inactive X chromosome and is excluded from autosomal material in cis.** *Hum Mol Genet* 1999, **8:**195-204.

3. Nasir J, Fisher EM, Brockdorff N, Disteche CM, Lyon MF, Brown SD: **Unusual molecular characteristics of a repeat sequence island within a Giemsa-positive band on the mouse X chromosome.** *Proc Natl Acad Sci U S A* 1990, **87:**399-403.

4. Shi YP, Mohapatra G, Miller J, Hanahan D, Lander E, Gold P, Pinkel D, Gray J: **FISH probes for mouse chromosome identification.** *Genomics* 1997, **45:**42-47.

5. Lawrence JB, Singer RH, Marselle LM: **Highly localized tracks of specific transcripts within interphase nuclei visualized by in situ hybridization.** *Cell* 1989, **57:**493-502.

6. Clerc P, Avner P: **Role of the region 3' to Xist exon 6 in the counting process of X-chromosome inactivation.** *Nat Genet* 1998, **19:**249-253.

7. de Napoles M, Mermoud JE, Wakao R, Tang YA, Endoh M, Appanah R, Nesterova TB, Silva J, Otte AP, Vidal M, et al: **Polycomb group proteins Ring1A/B link ubiquitylation of histone H2A to heritable gene silencing and X inactivation.** *Dev Cell* 2004, **7:**663-676.

8. Irizarry RA, Bolstad BM, Collin F, Cope LM, Hobbs B, Speed TP: **Summaries of Affymetrix GeneChip probe level data.** *Nucleic Acids Res* 2003, **31:**e15.

9. Conti L, Pollard SM, Gorba T, Reitano E, Toselli M, Biella G, Sun Y, Sanzone S, Ying QL, Cattaneo E, Smith A: **Niche-independent symmetrical self-renewal of a mammalian tissue stem cell.** *PLoS Biol* 2005, **3:**e283.

10. Keller G, Kennedy M, Papayannopoulou T, Wiles MV: **Hematopoietic commitment during embryonic stem cell differentiation in culture.** *Mol Cell Biol* 1993, **13:**473-486.

11. Smyth GK: **Linear models and empirical bayes methods for assessing differential expression in microarray experiments.** *Stat Appl Genet Mol Biol* 2004, **3:**Article3.

12. Breiman L: *Classification and regression trees.* Chapman & Hall; 1984.

13. Strasser H, Weber C: **The asymptotic theory of permutation statistics.** *Mathematical Methods of Statistics* 1999, **8:**220-250.

14. Hothorn T, Hornik K, Zeileis A: **Unbiased Recursive Partitioning: A Conditional Inference Framework.** *Journal of Computational and Graphical Statistics* 2006, **15:**651-674.

15. Zhang X, Rodriguez I, Mombaerts P, Firestein S: **Odorant and vomeronasal receptor genes in two mouse genome assemblies.** *Genomics* 2004, **83:**802-811.

16. Marzluff WF, Gongidi P, Woods KR, Jin J, Maltais LJ: **The human and mouse replication-dependent histone genes.** *Genomics* 2002, **80:**487-498.
